# Overexpression of Mitochondria Mediator Gene *TRIAP1* by miR-320b Loss Is Associated with Progression in Nasopharyngeal Carcinoma

**DOI:** 10.1371/journal.pgen.1006183

**Published:** 2016-07-18

**Authors:** Yingqin Li, Xinran Tang, Qingmei He, Xiaojing Yang, Xianyue Ren, Xin Wen, Jian Zhang, Yaqin Wang, Na Liu, Jun Ma

**Affiliations:** Sun Yat-sen University Cancer Center, State Key Laboratory of Oncology in South China, Collaborative Innovation Center of Cancer Medicine, Guangzhou, Guangdong, China; Dana Farber Cancer Institute, UNITED STATES

## Abstract

The therapeutic strategy for advanced nasopharyngeal carcinoma (NPC) is still challenging. It is an urgent need to uncover novel treatment targets for NPC. Therefore, understanding the mechanisms underlying NPC tumorigenesis and progression is essential for the development of new therapeutic strategies. Here, we showed that TP53-regulated inhibitor of apoptosis (TRIAP1) was aberrantly overexpressed and associated with poor survival in NPC patients. TRIAP1 overexpression promoted NPC cell proliferation and suppressed cell death *in vitro* and *in vivo*, whereas TRIAP1 knockdown inhibited cell tumorigenesis and enhanced apoptosis through the induction of mitochondrial fragmentation, membrane potential alteration and release of cytochrome *c* from mitochondria into the cytosol. Intersecting with our previous miRNA data and available bioinformatic algorithms, miR-320b was identified and validated as a negative regulator of TRIAP1. Further studies showed that overexpression of miR-320b suppressed NPC cell proliferation and enhanced mitochondrial fragmentation and apoptosis both *in vitro* and *in vivo*, while silencing of miR-320b promoted tumor growth and suppressed apoptosis. Additionally, TRIAP1 restoration abrogated the proliferation inhibition and apoptosis induced by miR-320b. Moreover, the loss of miR-320b expression was inversely correlated with TRIAP1 overexpression in NPC patients. This newly identified miR-320b/TRIAP1 pathway provides insights into the mechanisms leading to NPC tumorigenesis and unfavorable clinical outcomes, which may represent prognostic markers and potential therapeutic targets for NPC treatment.

## Author Summary

The therapeutic strategy for advanced nasopharyngeal carcinoma (NPC) is still challenging. The most urgent need for NPC is novel treatment targets. Therefore, understanding the mechanisms underlying NPC tumorigenesis and progression is essential for the development of new therapeutic strategies. Here, we identified TRIAP1 could serve as a prognostic biomarker in NPC, and function as an oncogene in NPC tumorigenesis and mitochondrial apoptosis through inhibiting the release of cytochrome *c*. Moreover, miR-320b post-transcriptionally regulated TRIAP1 expression, and exhibited inhibitory effects on proliferation and promoted apoptosis through targeting TRIAP1. Thus, our study provides new insights into the mechanisms of NPC tumorigenesis and progression and identifies novel therapeutic targets for NPC treatment.

## Introduction

Nasopharyngeal carcinoma (NPC) is the most prevalent head and neck malignancy in Southeast Asia, especially in Southern China [1]. A majority of NPC patients are diagnosed at advanced stages, leading to approximately 30% of NPC patients developing treatment failure [2]. Although NPC is a heterogeneous disease, a combination of radiotherapy and platinum-based chemotherapy remains the standard treatment method [3,4]. Therefore, identification of effective molecules regulating NPC development and progression is essential for developing novel therapeutic strategies.

Sustaining proliferative signaling and resisting apoptosis are typical hallmarks of cancer [5]. Mitochondria are at the core of programmed cell death or apoptosis [6,7]. Proteins involved in mitochondrial network could regulate the apoptotic pathway [8–10]. Thus, it is crucial to elucidate the molecular mechanisms of proliferation and mitochondrial apoptosis to excavate potential therapeutic targets for NPC therapy. TP53-regulated inhibitor of apoptosis (TRIAP1) is a small ~9-kDa protein transcriptionally activated by TP53 [11,12]. It has been reported that TRIAP1 protects cancer cells from apoptosis through interaction with hear shock protein 70–4 (HSP70) or the repression of cyclin-dependent kinase inhibitor 1 (p21) [12,13]. Recent evidence has also revealed that TRIAP1 contributes to the resistance of apoptosis in a mitochondria-dependent manner [14,15]. However, the function and clinical value of TRIAP1 remain unknown in NPC. In addition, TP53 is commonly inactivated in tumor cells to escape apoptosis, indicating there may be other mechanisms regulating TRIAP1 expression and extensive investigation is warrant.

MicroRNAs (miRNAs) are a class of small non-coding RNAs that negatively regulate gene expression by provoking mRNA degradation or suppressing mRNA translation [16–18]. Importantly, miRNAs have important roles in a wide range of biological processes, including cell proliferation, cell death and motility [19–21]. Accumulating evidences have shown that miRNAs are dysregulated and function as either oncogenes or tumor suppressors in different cancer types [22–24]. In our previous microarray study, a profile of deregulated miRNAs is identified in NPC [25,26], and some miRNAs affect cell growth, proliferation and metastasis in NPC [27–29]. However, it is yet unclear whether these miRNAs maintain their apoptotic effects in NPC. Therefore, understanding the role of miRNAs in apoptosis may provide insight into the mechanisms underlying carcinogenesis and aggressiveness in NPC.

In the present study, we demonstrated that TRIAP1 functioned as an oncogene in proliferation and apoptosis through preventing mitochondrial fragmentation and cytochrome *c* release and its overexpression was correlated with poor survival in NPC patients. miR-320b was revealed to negatively regulate TRIAP1 and exhibited proliferative inhibition and apoptotic promotion, which could be rescued by TRIAP1 overexpression. Thus, the altered miR-320b/TRIAP1 pathway contributes to the proliferation and apoptosis of NPC and may provide novel therapeutic targets for NPC treatment.

## Results

### TRIAP1 is upregulated and correlates with poor survival in NPC patients

**Fig 1:**
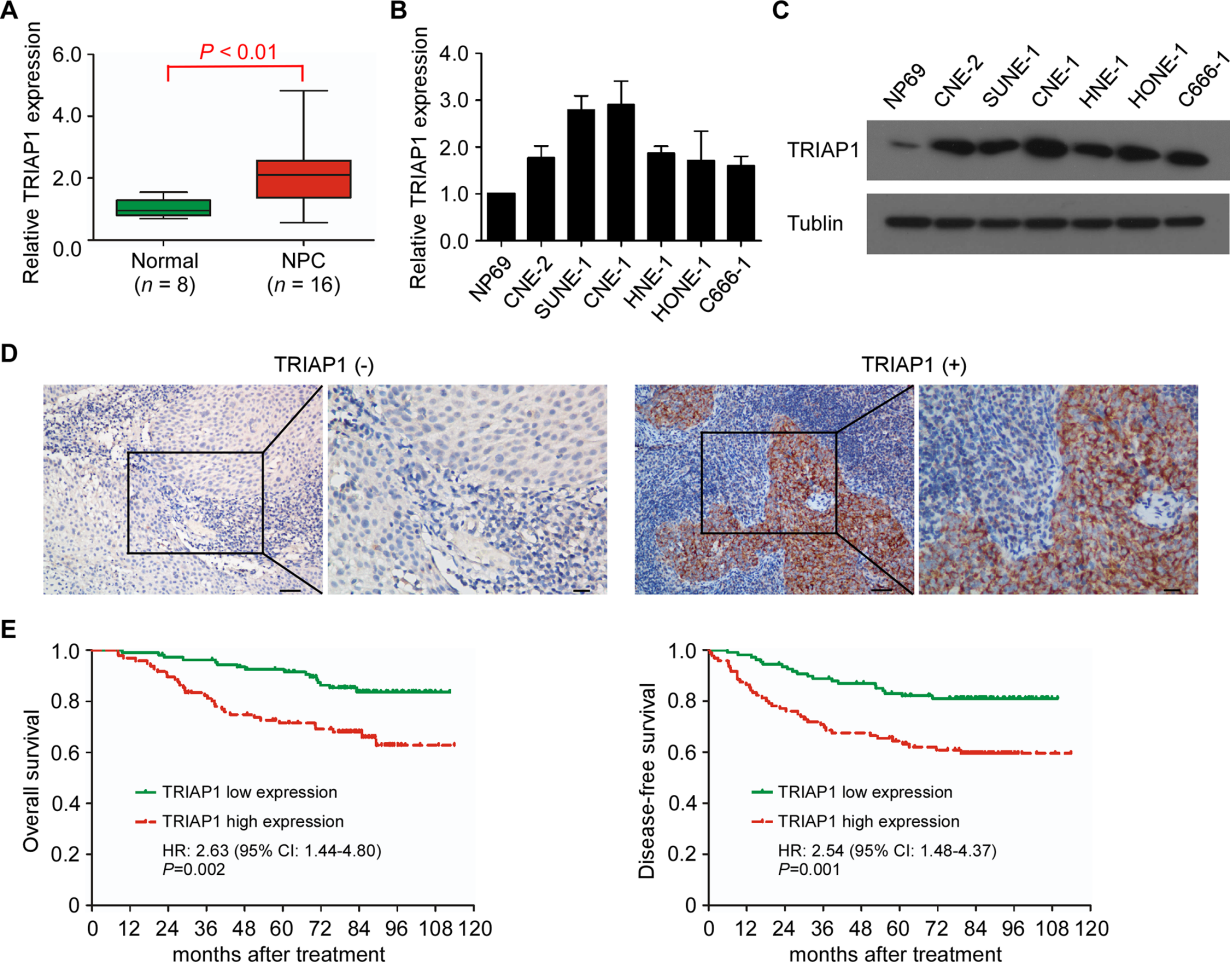
Upregulation of TRIAP1 correlates with poor survival in NPC patients. **A** and **B**, Quantitative RT-PCR analysis of TRIAP1 expression in normal nasopharyngeal epithelial tissues (*n* = 8) and NPC (*n* = 16) (**A**) and in immortalized NP69 cells and NPC cell lines (**B**). U6 was used as an endogenous control. Each experiment was independently repeated at least three times. The data are presented as the mean ± s.d. The *P* value was determined by Student’s *t*-test. (**C**) Representative western blotting analysis of TRIAP1 expression in immortalized NP69 cells and NPC cell lines. α-Tubulin served as a loading control. The experiment was independently repeated at least three times. (**D**) Representative immunohistochemistry (IHC) staining with low and high TRIAP1 expression. Scale bar, left panel 50 μm; right panel 20 μm. (**E**) Kaplan-Meier analysis of overall survival (left panel) and disease-free survival (right panel) for NPC patients with low (*n* = 108) versus high (*n* = 96) expression of TRIAP1. *P* value was determined using the log-rank test.

To investigate the clinical significance of TRIAP1 in NPC, we first examined TRIAP1 mRNA expression in 16 fresh-frozen NPC and 8 normal nasopharyngeal epithelial tissues. The mRNA expression level of TRIAP1 was significantly upregulated in NPC tissues (Fig 1A, *P* < 0.01) and in 6 NPC cell lines compared with the normal nasopharyngeal epithelial cell line NP69 (Fig 1B). In addition, protein immunoblotting analysis confirmed high TRIAP1 expression in various NPC cell lines (Fig 1C).

To further evaluate the expression status of TRIAP1 in NPC, we performed immunohistochemistry (IHC) for TRIAP1 in 204 NPC specimens. The results showed that TRIAP1 was overexpressed in 47.1% (96/204) of the NPC specimens (Fig 1D). Importantly, the level of TRIAP1 expression was strongly correlated with distant metastasis (*P* < 0.001) and death (*P* = 0.003; S1 and S2 Tables). Patients with high TRIAP1 expression showed significantly shorter 5-year overall survival (OS; 92.5% vs. 71.5%, *P* = 0.002) and disease-free survival (DFS; 83.1% vs. 71.5%, *P* = 0.001; Fig 1E) rates than those with low TRIAP1 expression. Moreover, multivariate analysis revealed TRIAP1 overexpression was an independent prognostic factor for OS (HR, 2.75; 95% CI, 1.50–5.03; *P* = 0.001) and DFS (HR, 2.54; 95% CI, 1.47–4.38; *P* < 0.001; S3 Table). Taken together, these data demonstrate that TRIAP1 overexpression is a risk factor for a poor prognosis in NPC patients.

### TRIAP1 promotes NPC cell proliferation and regulates apoptosis *in vitro*

**Fig 2:**
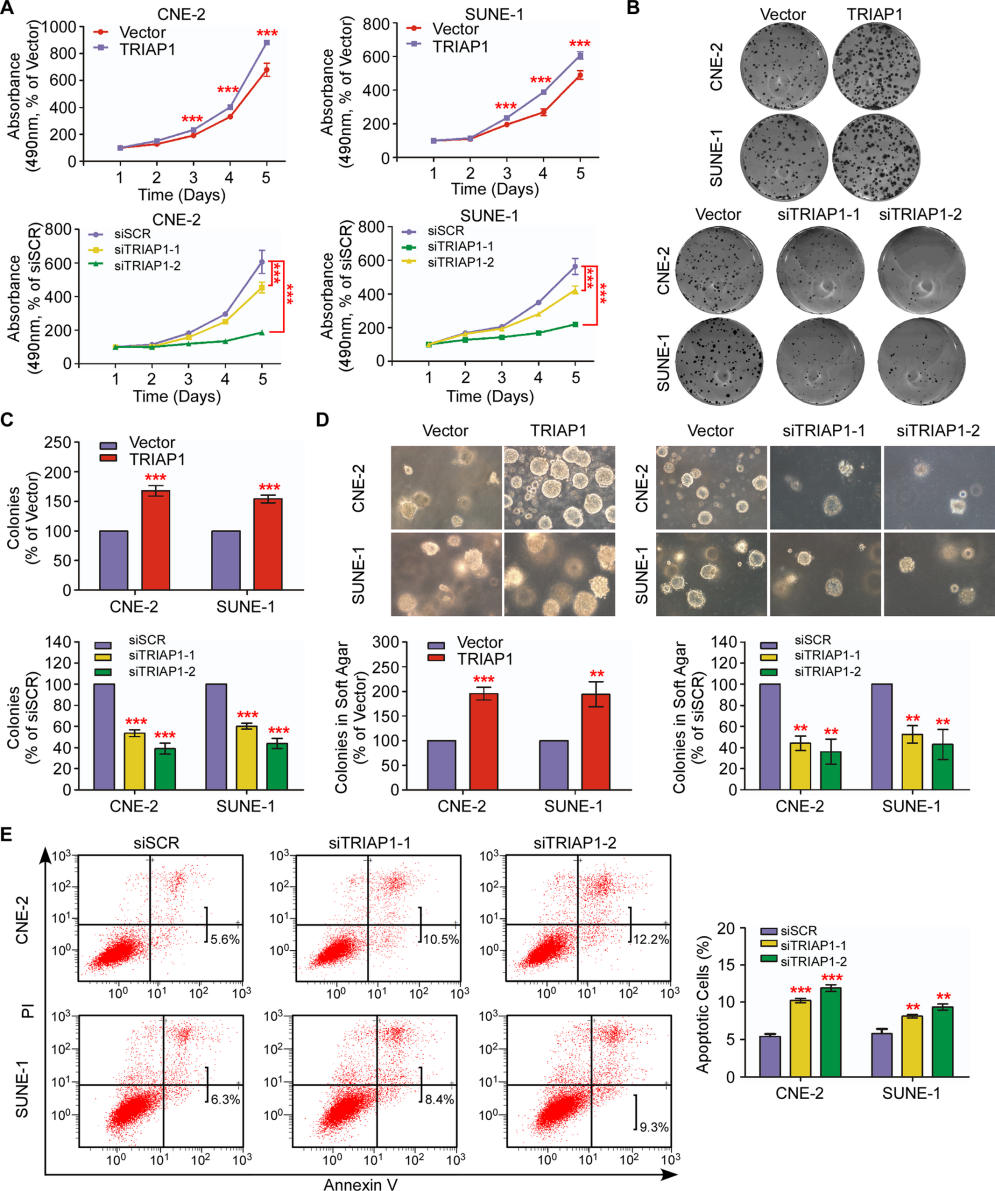
TRIAP1 promotes NPC cell proliferation and suppresses apoptosis *in vitro*. (**A**) MTT assay for CNE-2 (left panel) and SUNE-1 (right panel) cells transiently transfected with an empty vector or TRIAP1 (upper panel); or scrambled siRNA control (siSCR) or TRIAP1-specific siRNA (siTRIAP1-1, siTRIAP1-2) (lower panel). (**B** and **C**) Representative images (**B**) and quantification (**C**) of the colony-formation assays for the above-mentioned NPC cells. (**D**) Representative images (upper panel) and quantification (lower panel) of the colony-formation assays for the above-mentioned NPC cells. (**E**) Representative dot plots (left panel) and quantification (right panel) of the flow cytometric analysis of TRIAP1 knockdown in CNE-2 and SUNE-1 cells subjected to Annexin V and propidium iodide (PI) staining. Each experiment was independently repeated at least three times. The data are presented as the mean ± s.d. Student’s *t*-test, ** *P* < 0.01, *** *P* < 0.001.

To explore the biological role of TRIAP1 in NPC, we transiently overexpressed or knocked down TRIAP1 in CNE-2 and SUNE-1 cells (S1A–S1D Fig). While cell proliferation was significantly promoted following ectopic TRIAP1 overexpression, TRIAP1 knockdown remarkably inhibited cell proliferation (Fig 2A, *P* < 0.001). Furthermore, TRIAP1 overexpression significantly increased the colony-formation rate and anchorage-independent growth ability, which were impaired by TRIAP1 silencing (Fig 2B–2D, *P* < 0.01). These data suggest that TRIAP1 promotes NPC cell proliferation.

Furthermore, we investigated the effect of TRIAP1 on apoptosis through flow cytometric analysis and found that knockdown of TRIAP1 expression induced a significantly higher rate of apoptotic cells compared with the control group (Fig 2E, *P* < 0.01). These findings demonstrate that TRIAP1 participates in regulating NPC cell apoptosis.

### TRIAP1 promotes NPC tumorigenesis and suppresses cell apoptosis *in vivo*

**Fig 3:**
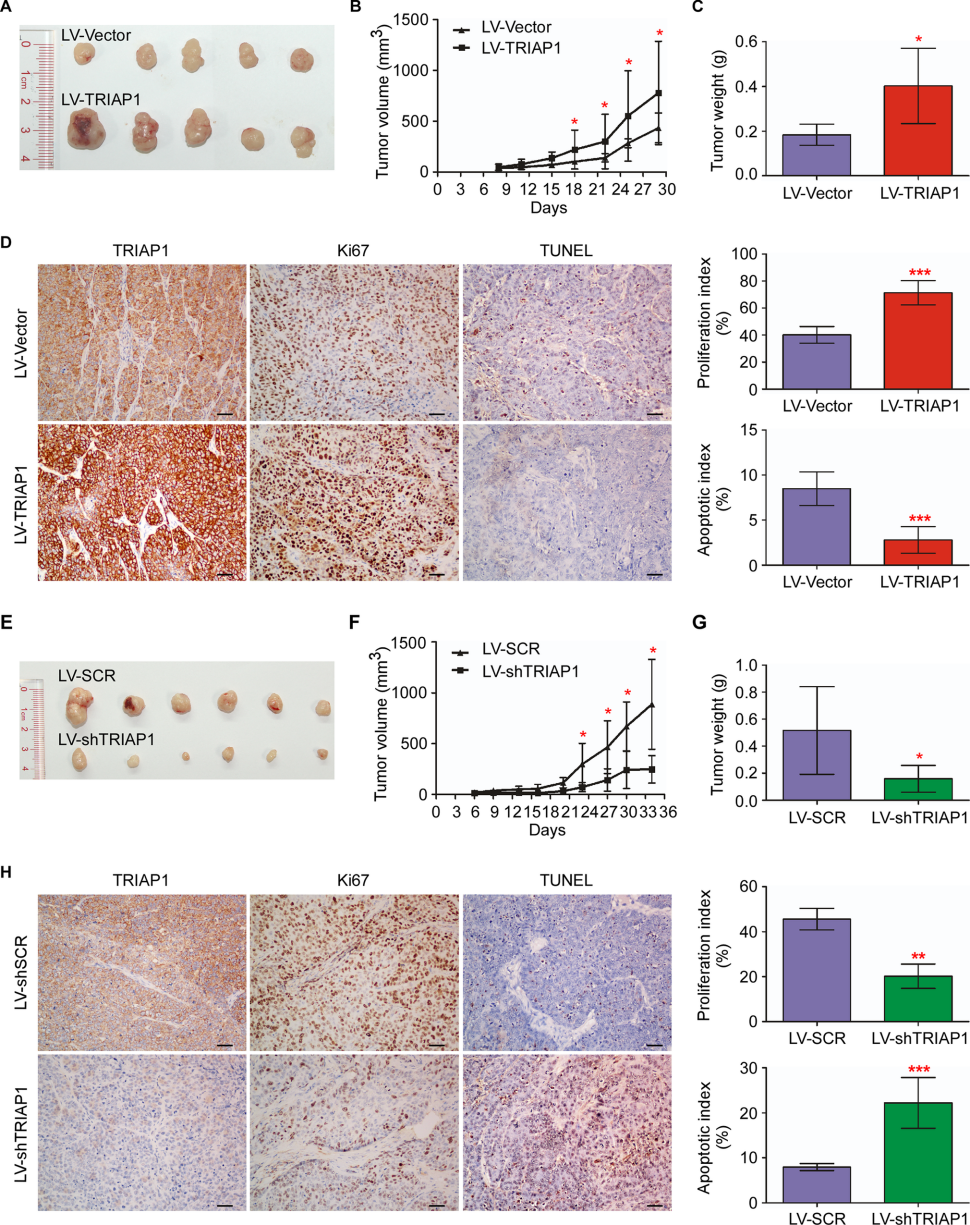
TRIAP1 promotes NPC cell growth and inhibits apoptosis *in vivo*. (**A-C**) Representative images (**A**), tumor volume growth curves (**B**) and weight (**C**) of tumors developed in xenograft models subcutaneously injected with SUNE-1 cells stably expressing the empty vector or overexpressing TRIAP1. (**D**) Representative images (left panel) and quantification of the percentage (right panel) of Ki67-positive and TUNEL-positive cells in xenografts. Scale bar, 50 μm. (**E-G**) Representative images (**E**), tumor volume growth curves (**F**) and weight (**G**) of tumors developed in xenograft models subcutaneously injected with SUNE-1 cells stably expressing the scrambled control (SCR) or TRIAP1-specific shRNA (shTRIAP1). (**H**) Representative images (left panel) and quantification of percentage (right panel) of Ki67-positive and TUNEL-positive cells in xenografts. Scale bar, 20 μm. The data are presented as the mean ± s.d. Student’s *t*-test, * *P* < 0.05, ** *P* < 0.01, *** *P* < 0.01.

Next, we investigated the effect of TRIAP1 on tumorigenesis *in vivo* through xenograft tumor models. As shown in Fig 3A–3C, TRIAP1 overexpression significantly enhanced tumor growth, with regard to both tumor volume and tumor weight, compared with the control LV-Vector group. TRIAP1 expression in dissected specimens was confirmed by IHC (Fig 3D). In addition, TRIAP1 overexpression displayed a higher proportion of Ki67-positive cells and a lower percentage of TdT-mediated dUTP Nick-End Labeling (TUNEL)-positive cells (Fig 3D, *P* < 0.001), suggesting that cells with ectopic TRIAP1 expression were actively proliferating. Conversely, tumor growth, tumor size and tumor weight were significantly inhibited by TRIAP1 knockdown (Fig 3E–3G, *P* < 0.05). Meanwhile, the TRIAP1-knockdown group showed a decreased proliferation index and increased apoptotic index (Fig 3H, *P* < 0.01). All together, these results support that TRIAP1 promotes NPC tumor growth and inhibits cell apoptosis *in vivo*.

### TRIAP1 regulates mitochondrial apoptosis

**Fig 4:**
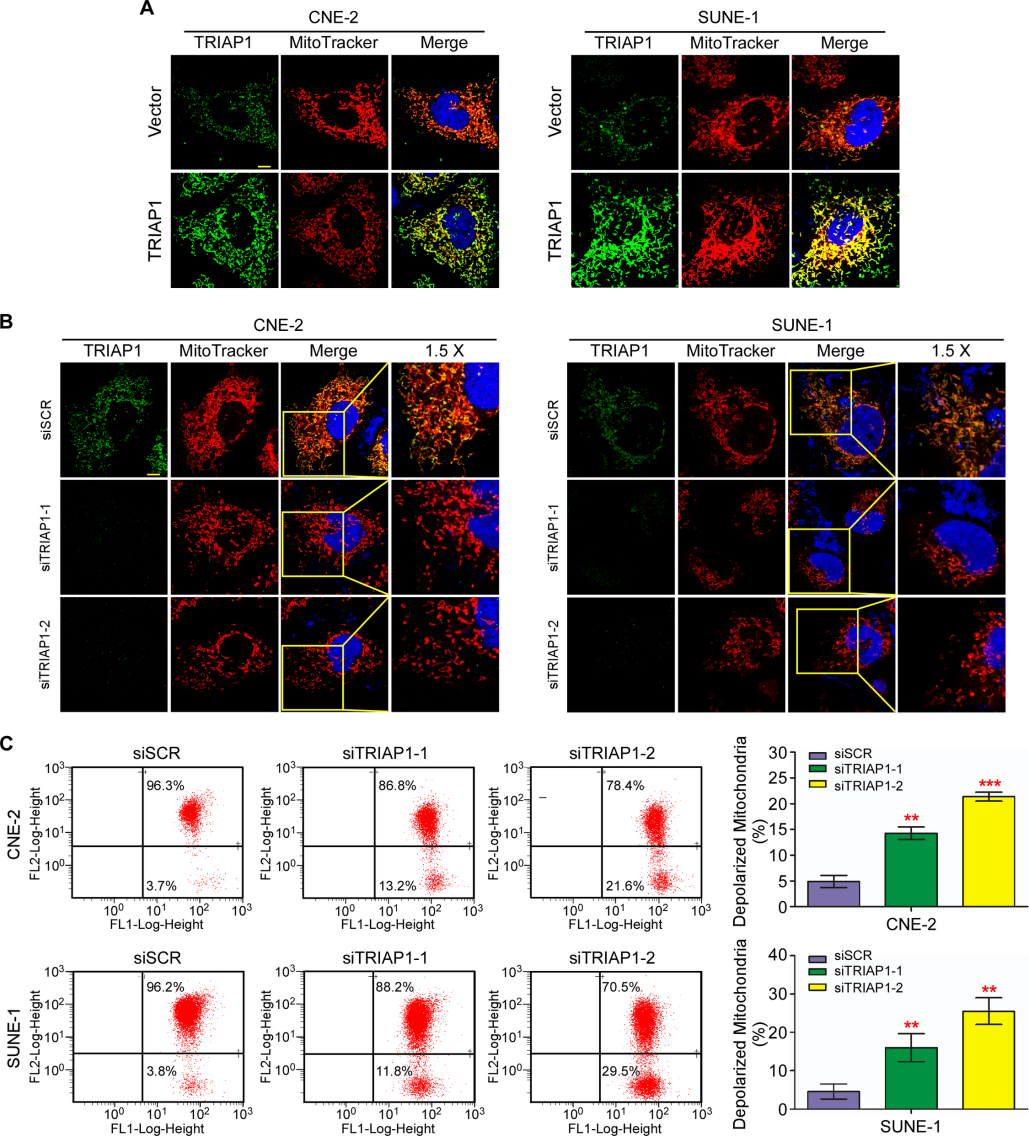
TRIAP1 regulates mitochondrial fragmentation and membrane potential. **A** and **B**, Representative images of mitochondria and TRIAP1 subcellular location for CNE-2 (left panel) and SUNE-1 (right panel) cells transiently transfected with an empty vector (upper panel) or TRIAP1 (lower panel) (**A**); or siSCR, siTRIAP1-1 or siTRIAP1-2 (**B**) after stained with MitoTracker Red and TRIAP1 primary antibody. Scale bar, 10 μm. (**C**) Representative dot plots (left panel) and quantification (right panel) of mitochondrial membrane potential of TIRAP1 knockdown in CNE-2 and SUNE-1 cells subjected to JC-1 staining. The percentage of cells with FL1-positive and FL2-negative signals represents depolarized mitochondrial cells. Each experiment was independently repeated at least three times. The data are presented as the mean ± s.d. Student’s *t*-test, ** *P* < 0.01, *** *P* < 0.001.

To explore the underlying mechanism of TRIAP1 on NPC cell proliferation and apoptosis, we investigated the subcellular location of TRIAP1. The observation showed that ectopically expressed TRIAP1 accumulated in the mitochondria, indicating that TRIAP1 co-localized with mitochondria (Fig 4A). Furthermore, TRIAP1 knockdown led to marked mitochondrial fragmentation in both live mitochondrial images (S2 Fig) and fixed mitochondrial views (Fig 4B). We next investigated the status of mitochondrial membrane potential (△*Ψ*m). TRIAP1 knockdown induced a significantly increased number of depolarized mitochondria (Fig 4C). Taken together, these findings indicate that TRIAP1 participates in the regulation of mitochondrial fragmentation and is required for normal polarized mitochondrial membrane potential.

### TRIAP1 regulates mitochondria-dependent apoptosis through cytochrome *c* release

**Fig 5:**
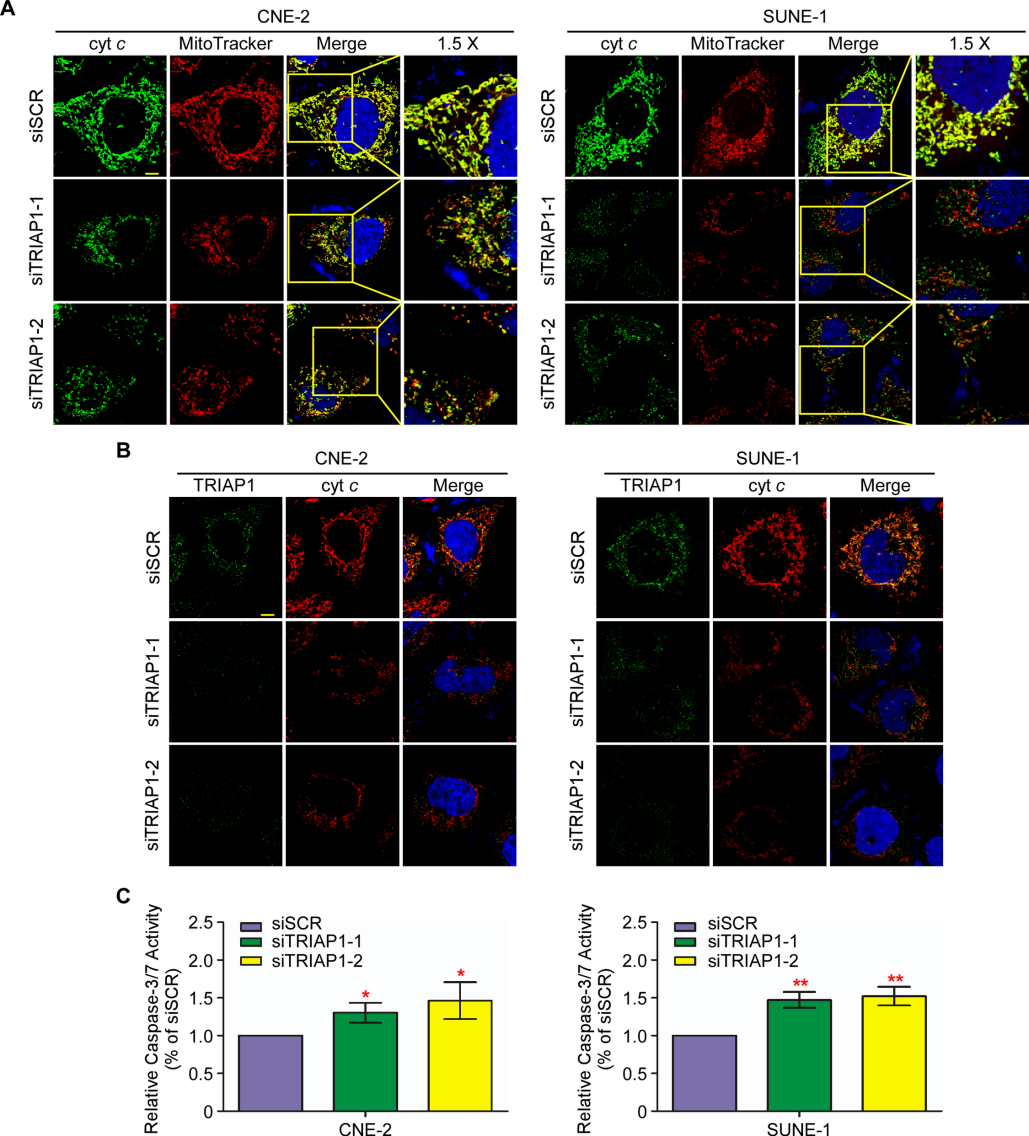
TRIAP1 regulates apoptosis through controlling the release of cytochrome *c*. **A** and **B**, Release of cytochrome *c* is increased upon TRIAP1 knockdown. Representative images of mitochondria, cytochrome *c* (**A**) and TRIAP1 (**B**) subcellular locations for CNE-2 (left panel) and SUNE-1 (right panel) cells transiently transfected with siSCR, siTRIAP1-1 or siTRIAP1-2 after staining with MitoTracker Red, cytochrome *c* or TRIAP1 primary antibodies. Scale bar, 10 μm. (**C**) Caspase-3/7 assay for cells with TRIAP1 knockdown. Each experiment was independently repeated at least three times. The data are presented as the mean ± s.d. Student’s *t*-test, * *P* < 0.05, ** *P* < 0.01.

Furthermore, we examined the potential role of TRIAP1 in mitochondria-dependent apoptosis. Immunofluorescent staining displayed that cytochrome *c* co-localized with mitochondria and TRIAP1 (Fig 5A and 5B). Interestingly, the loss of TRIAP1 induced the release of cytochrome *c* from mitochondria into the cytosol accompanied by mitochondrial fragmentation (Fig 5A and 5B). Subsequently, the activity of caspase-3 and -7 was significantly increased by TRIAP1 knockdown, revealing a significant induction of apoptosis (Fig 5C). Together, these results suggest that knockdown of TRIAP1 led to apoptosis through mitochondrial fragmentation and the subsequent release of cytochrome *c* from mitochondria.

### miR-320b directly targets TRIAP1

**Fig 6:**
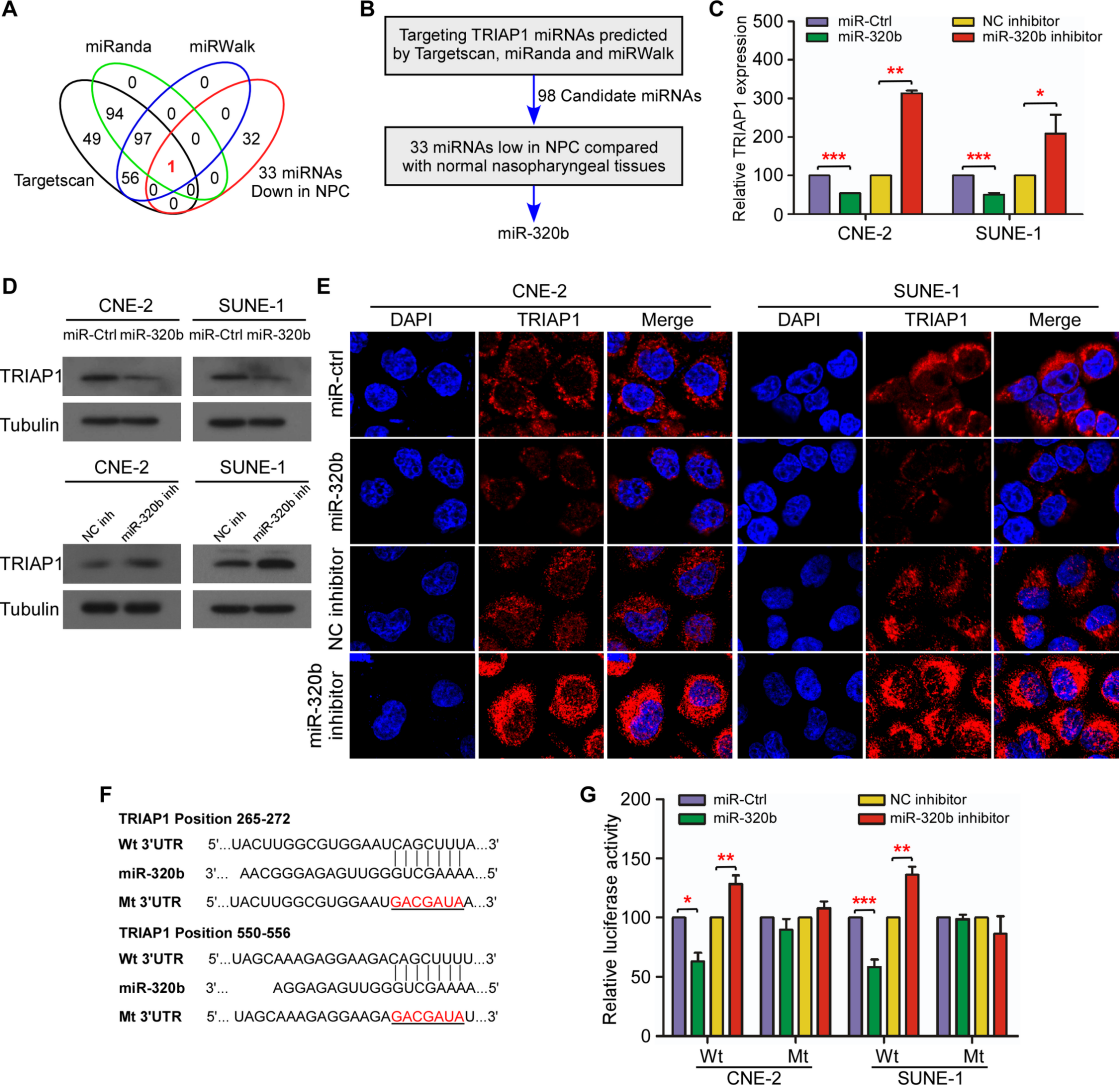
miR-320b negatively regulates TRIAP1 in NPC. (**A**) Schematic diagram of candidate miRNAs predicted by three different bioinformatics algorithms. Each labeled circle represents one algorithm with the number of its predicted miRNAs. (**B**) Outline of miRNA screening procedure. (**C-E**) Quantification of TRIAP1 mRNA expression by quantitative RT-PCR (**C**) and TRIAP1 protein expression by western blotting (**D**) and immunofluorescent staining (**E**) in CNE-2 and SUNE-1 cells after transient transfection with miR-320b mimic or miR-320b inhibitor or control. (**F**) Predicted miR-320b target sequence (Wild-type: Wt) or mutant sequence (Mt) in the 3′ UTR of TRIAP1. (**G**) Luciferase assay of CNE-2 and SUNE-1 cells after co-transfection with wild-type (Wt) or mutant (Mt) MET 3′ UTR reporter genes and miR-320b mimic miR-320b inhibitor or control. Each experiment was independently repeated at least three times. The data are presented as the mean ± s.d. Student’s *t*-test, ** *P* < 0.01, *** *P* < 0.001.

To investigate the mechanisms of TRIAP1 expression aberration, we used available bioinformatic algorithms as filters to screen miRNAs targeting TRIAP1. A total of 98 miRNAs were identified as candidates and subsequently intersected with the 33 downregulated miRNAs identified in our published data set (NCBI/GEO/GSE32960, *n* = 330, including 312 NPC tissues and 18 normal nasopharyngeal tissues; Fig 6A and 6B and S3 Fig) [25]. Finally, miR-320b was identified as the sole candidate (Fig 6B, *P* < 0.01). In determining whether miR-320b negatively regulates TRIAP1 expression, we found that miR-320b mimics significantly inhibited TRIAP1 expression at both the mRNA and protein levels, whereas miR-320b inhibitor increased its expression in NPC cells (Fig 6C–6E, *P* < 0.05). To further confirm the site-specific repression of miR-320b on TRIAP1, we constructed wild-type and mutant TRIAP1 3′ UTR luciferase reporter vectors (Fig 6F). miR-320b overexpression or inhibition suppressed or increased the luciferase activity of the wild-type TRIAP1 3′ UTR reporter gene but had no inhibitory effect on the mutant reporter (Fig 6G, *P* < 0.05). Taken together, these data demonstrate that TRIAP1 is a novel direct target of miR-320b in NPC cells.

### miR-320b exerts its functions on cell proliferation, mitochondrial function and apoptosis through targeting TRIAP1 in NPC

**Fig 7:**
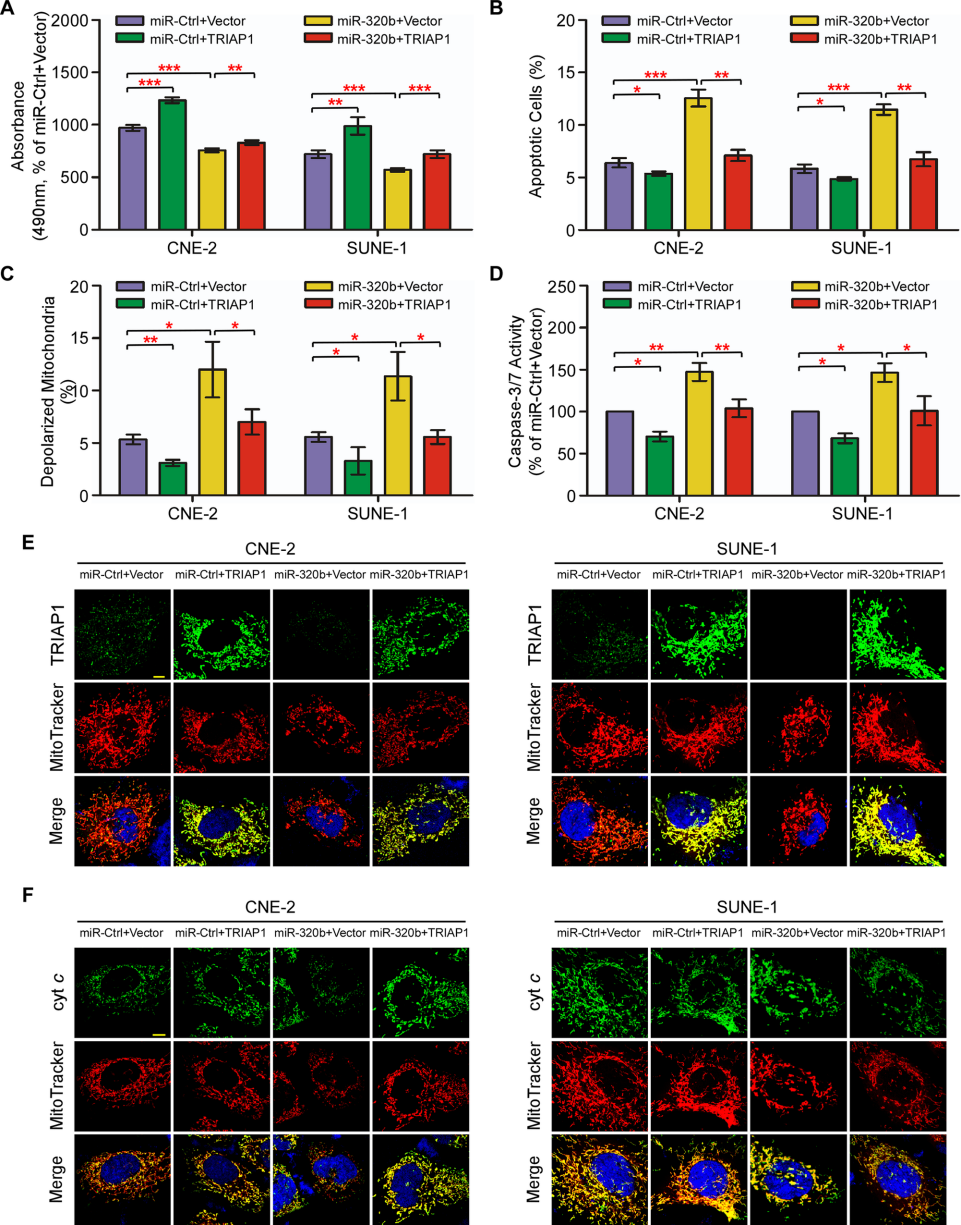
TRIAP1 mediates the effects of miR-320b on NPC cell proliferation, mitochondrial fragmentation and apoptosis. (**A-F**) CNE-2 and SUNE-1 cells were co-transfected with a miR-320b mimic or miR-Ctrl and either the empty vector (Vector) or plasmid overexpressing TRIAP1. (**A**) MTT assay showing that recovery of TRIAP1 partially rescues the inhibitory effects of miR-320b on cell proliferation. (**B-F**) Flow cytometric analysis (**B-C**), caspase-3/7 (**D**) and immunofluorescent staining (**E-F**) assay showing that restoration of TRIAP1 reverses the promoting effects of miR-320b on mitochondrial fragmentation, apoptosis and cytochrome *c* release from mitochondria. Scale bar, 10 μm. Each experiment was independently repeated at least three times. The data are presented as the mean ± s.d. Student’s *t*-test, * *P* < 0.05, ** *P* < 0.01, *** *P* < 0.001.

Subsequently, we investigated whether miR-320b has biological roles in NPC progression. Similar to the effect induced by loss of TRIAP1, overexpression of miR-320b significantly suppressed cell proliferation (Fig 7A, *P* < 0.01), but led to mitochondrial membrane depolarization, mitochondrial fragmentation and apoptosis (Fig 7B–7E and S4A and S4B Fig, *P* < 0.05). Inversely, miR-320b inhibition increased cell proliferation, but decreased mitochondrial membrane depolarization and apoptosis (S5A–S5F Fig, *P* < 0.05). Furthermore, we explored how miR-320b exerts its functional effects. Restoration of TRIAP1 remarkably abrogated the proliferation inhibition, mitochondrial membrane depolarization, fragmentation and apoptosis induced by miR-320b (Fig 7A–7E and S4A and S4B Fig, *P* < 0.05), while inhibition of TRIAP1 expression significantly abrogated the induction of proliferation, and the suppression of mitochondrial membrane depolarization and apoptosis induced by miR-320b knockdown (S5A–S5F Fig, *P* < 0.05). In addition, enforced TRIAP1 overexpression prevented cytochrome *c* release from mitochondria to the cytoplasm (Fig 7F and S6A and S6B Fig). These results suggest that TRIAP1 is a functional mediator of miR-320b on cell proliferation and mitochondria-dependent apoptosis in NPC.

### Loss of miR-320b is correlated with TRIAP1 overexpression and tumorigenesis in NPC

**Fig 8:**
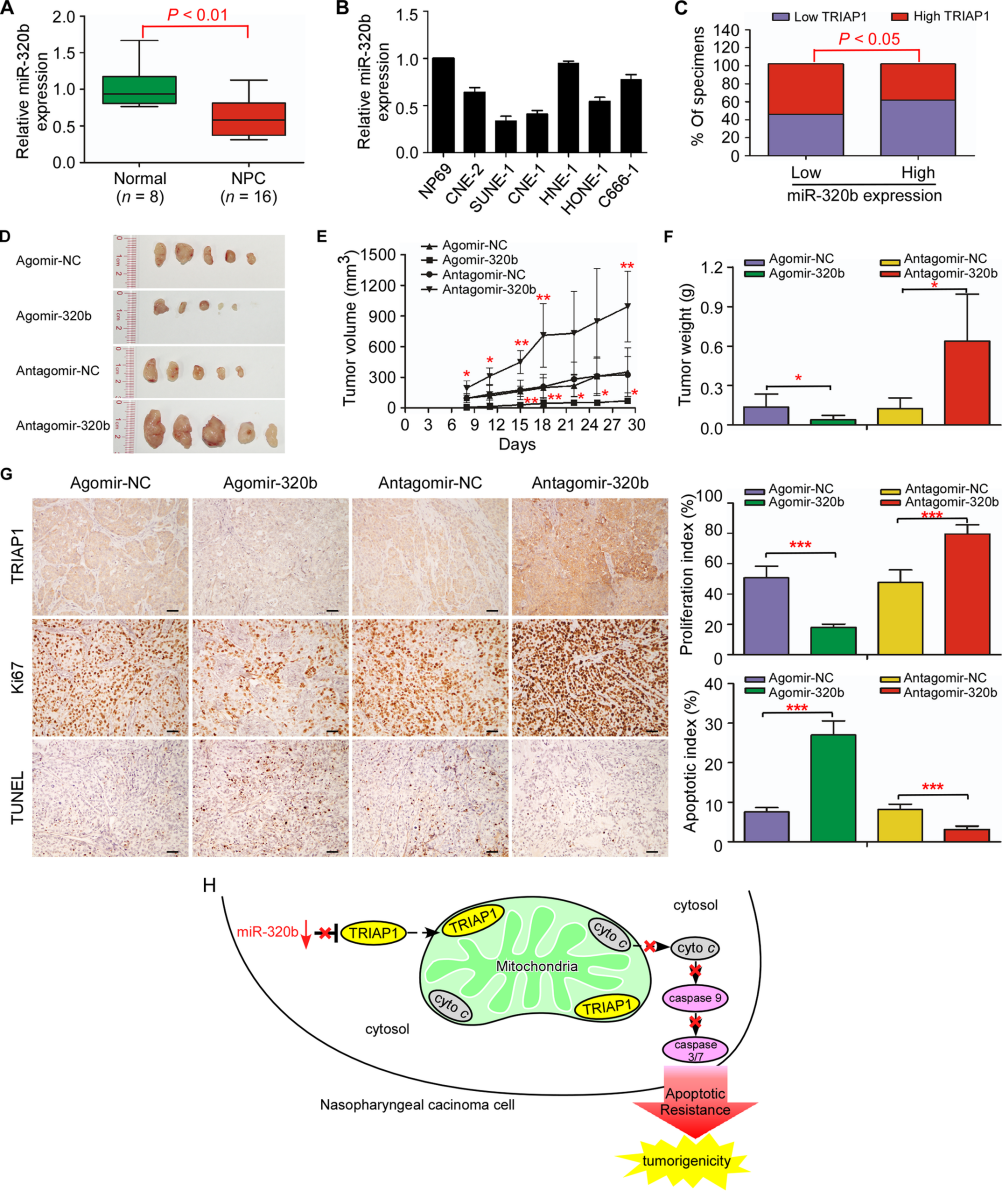
Loss of miR-320b is associated with TRIAP1 overexpression and tumor growth in NPC. **A** and **B**, quantitative RT-PCR analysis of miR-320b expression in normal nasopharyngeal epithelial tissues (*n* = 8) and NPC (*n* = 16) (**A**); and in immortalized NP69 cells and NPC cell lines (**B**). U6 was used as an endogenous control. Each experiment was independently repeated at least three times. The data are presented as the mean ± s.d. Student’s *t*-test. **C**, percentages of specimens showing low or high miR-320b expression relative to TRIAP1 expression level. Statistical significance was determined using the Pearson’s Chi-Square test. (**D-G**) SUNE-1 cells were subcutaneously injected into the dorsal flank of nude mice after pre-transfected with agomir-320b, antagomir-320b or NC control (100nM). Intratumoral injection of agomir-320b, antagomir-320b or NC control was performed after tumor formation. (**D-F**) Representative images (**D**), tumor volume growth curves (**E**) and weight (**F**) of tumors developed in xenograft models injected with agomir-320b, antagomir-320b or NC control. (**G**) Representative images (left panel) and quantification of the percentage (right panel) of Ki67-positive and TUNEL-positive cells in xenografts. Scale bar, 50 μm. The data are presented as the mean ± s.d. Student’s *t*-test, * *P* < 0.05, ** *P* < 0.01, *** *P* < 0.01. **H**, proposed model. Downregulated miR-320b determined TRIAP1 overexpression to prevent mitochondrial fragmentation and cytochrome *c* release into the cytosol, leading to apoptosis resistance and tumorigenesis in NPC.

Consistent with our previous miRNA microarray data (NCBI/GEO/GSE32960; S7 Fig), miR-320b was significantly downregulated in 16 fresh-frozen NPC compared with 8 normal nasopharyngeal epithelial tissues, as well as in 6 NPC cell lines compared with the normal cell line NP69 (Fig 8A and 8B). Quantitative RT-PCR revealed that miR-320b expression was inversely correlated with TRIAP1 levels in NPC tissues (*n* = 204; Fig 8C; *P* < 0.01). When combining the expression of miR-320b and TRIAP1, patients in group III with low miR-320b expression and high TRIAP1 expression displayed worse OS and DFS than those in groups I and II with low TRIAP1 expression (*n* = 204; S8A and S8B Fig; *P* < 0.001). Furthermore, miR-320b overexpression significantly suppressed tumor growth and displayed a lower proliferation index and a higher apoptotic index, while miR-320b inhibition promoted tumorigenesis and inhibited apoptosis *in vivo* (Fig 8D–8G, S9 Fig; *P* < 0.05). Taken together, these results support that the miR-320b/TRIAP1 pathway regulates the proliferation and apoptosis by repressing the release of cytochrome *c* from mitochondria, leading to NPC tumorigenesis and poor clinical outcomes (Fig 8H).

## Discussion

In our current study, we found that TRIAP1 was upregulated and associated with poor clinical outcomes in NPC. Moreover, we firstly reported that TRIAP1 could be post-transcriptionally regulated by miR-320b, and TRIAP1 expression was inversely correlated with miR-320b expression in clinical NPC samples. Furthermore, miR-320b inhibited cell proliferation and increased apoptosis through the release of cytochrome *c* from mitochondria in a TRIAP1-dependent manner. Therefore, our findings uncovered a novel mechanism post-transcriptionally regulating TRIAP1 expression by miR-320b and its role in tumorigenesis and unfavorable survival in NPC.

Sustaining proliferation and resisting apoptosis are hallmarks of cancer [5]. Apoptosis is programmed cell death regulated by intrinsic and extrinsic pathways centralized in the mitochondria [6,7]. However, cell death is commonly resisted in cancer cells. Mitochondria constantly undergo fusion and fission, which are required for cells to maintain mitochondrial integrity and respond to intrinsic apoptotic stimuli [30–33]. A low fusion-to-fission ratio has been reported to result in the loss of mitochondrial fusion, the generation of mitochondrial fragmentation and the release of cytochrome *c* to trigger cell death (apoptosis) [8–10]. Proteins involved in mitochondrial fusion and fission may participate in cancer cell resistance to apoptotic stimuli and serve as new therapeutic targets. A number of studies have observed mitochondrial-mediated apoptosis in treating NPC cells [34,35]. However, the regulation of mitochondrial network dynamic and apoptosis in NPC remains undefined.

Emerging evidence indicates that TRIAP1 promotes cell survival and prevents apoptosis [11–14,36]. In our present study, we found that TRIAP1 promoted NPC cell proliferation and suppressed apoptosis *in vitro* and *in vivo*, supporting the contribution of TRIAP1 in NPC development and progression. Moreover, we demonstrated that TRIAP1 overexpression was associated with poor survival and was an independent risk factor in NPC, indicating a significant therapeutic implication of TRIAP1 in NPC. As we known, mitochondrial fragmentation is required for apoptosis induction. In this study, we found that knockdown of TRIAP1 induced mitochondrial fragmentation, membrane potential depolarization and the subsequent release of cytochrome *c*, and enhanced apoptosis in NPC cells, which is consistent with a previous study in colon cancer [14]. Although another study reports that TRIAP1 exerts its function through repressing p21 [13], we found that knockdown of TRIAP1 did not increased, but decreases p21 expression, suggesting that TRIAP1did not function through repressing p21 in NPC (S10A Fig). Our study elucidates the mechanisms of TRIAP1 regulating mitochondrial fragmentation and apoptosis in NPC, suggesting that TRIAP1 modulation can be a promising therapy for NPC apoptotic resistance.

As we known, TRIAP1 is transcriptionally upregulated by TP53 [7,8] and it has been reported that TP53 can be activated by EBV encoded protein LMP1 in NPC [37–39]. However, no obvious upregulation of TP53 and TRIAP1 was observed after LMP1 overexpression in NPC cells (S10B Fig), suggesting that TRIAP1 is not regulated though LMP1/TP53 pathway in NPC and there may be other regulatory mechanisms involved in TRIAP1 overexpression. In this study, we provided evidence that miR-320b negatively regulated TRIAP1 expression and exerted its function on mitochondrial fragmentation and apoptosis by targeting TRIAP1. The inhibitory effect of miR-320b is consistent with the previous study in other cancer types and cardiomyopathy [40–42]. Here, the miR-320b level was inversely correlated with TRIAP1 expression in NPC patients, revealing that loss of miR-320b determines TRIAP1 overexpression and function in NPC. We also acknowledged that the correlation between miR-320b and TRIAP1 expression was modest in NPC patients, which indicating that there may be some other mechanisms involved in regulating TRIAP1 expression.

In conclusion, our study revealed TRIAP1 as an oncogene in tumor progression and unfavorable survival, and miR-320b as a novel post-transcriptional regulator of TRIAP1 expression in NPC. The newly identified miR-320b/TRIAP1 pathway uncovers the molecular mechanisms underlying tumorigenesis and poor clinical outcomes of NPC and may facilitate the development of novel therapeutic strategies against NPC.

## Materials and Methods

### Ethics statement

For Human Subject Research, this study was approved by the Institutional Ethical Review Boards of Sun Yat-sen University Cancer Center (approval number: L20150201). Written informed consent was obtained from each patient before the study. For Animal Research, all experiments were performed according to the guidelines approved by the Institutional Animal Care and Use Ethics Committee of Sun Yat-sen University Cancer Center (approval number: 00111032).

### Clinical specimens

A total of 204 consecutive patients diagnosed with non-distant metastatic NPC were recruited from Sun Yat-sen University Cancer Center between January 2004 and January 2007. Paraffin-embedded biopsy specimens of individual patients were histologically-confirmed and collected for immunohistochemistry and quantitative RT-PCR. Written informed consent was obtained from each patient before the study. This study was approved by the Institutional Ethical Review Boards of Sun Yat-sen University Cancer Center. No patient had received radiotherapy or chemotherapy before biopsy. The TNM stage was reclassified according to the 7^th^ edition of the AJCC Cancer Staging Manual. All patients were treated with radiotherapy, as previously described [43]. Patients with stage III-IV NPC received concurrent platinum-based chemotherapy [3,44]. The median follow-up time was 81.7 months (range, 8.2 to 113.9 months). The detailed clinicopathological characteristics are listed in S1 Table. Sixteen fresh-frozen NPC samples with histological diagnosis and eight normal nasopharyngeal epithelium samples were collected and stored in liquid nitrogen until required.

### Immunohistochemistry

IHC analysis was performed on individual sections of 204 specimens, using the polyclonal anti-TRIAP1 antibody (1:200, Sigma-Aldrich, Ronkonkoma, NY, USA). The degree of immunostaining was independently evaluated by two pathologists blinded to the clinicopathological characteristics of the patients. The scores were determined on the basis of the staining intensity and the percentage of positively stained cells. The staining intensity was graded as follows: 0, no staining; 1, weak staining, light yellow; 2, moderate staining, yellow brown; and 3, strong staining, brown. The percentages were scored according to the following standard: 1, < 10% positive cells; 2, 10–35% positive cells; 3, 35–70% positive cells; and 4, > 70% positive cells, as previously described [45]. The staining index was used to evaluate TRIAP1 expression in NPC sections, with possible scores of 0, 1, 2, 3, 4, 6, 8, 9 and 12. Cutoff values were determined on the basis of Receiver operating characteristic (ROC) curve analysis, and classified as follow: low TRIAP1 expression, staining index < 6; and high TRIAP1 expression, staining index ≥ 6 [46,47].

### Cell culture

The human immortalized nasopharyngeal epithelial cell line NP69 and human NPC cell lines CNE-2, SUNE-1, CNE-1, HNE-1, HONE-1 and C666-1 were obtained from Professor Musheng Zeng within 6 months and authenticated by short tandem repeat profiling (Sun Yat-sen University, Guangzhou, China). NP69 was cultured in keratinocyte/serum-free medium (Invitrogen, Grand Island, NY, USA) supplemented with bovine pituitary extract (BD Biosciences, San Diego, CA, USA). CNE-2, SUNE-1, CNE-1, HNE-1, HONE-1 and C666-1 were grown in RPMI-1640 (Invitrogen) supplemented with 10% FBS (Gibco, Grand Island, NY, USA); 293FT cells were maintained in DMEM (Invitrogen) supplemented with 10% FBS.

### RNA extraction, reverse transcription and quantitative RT-PCR

Total RNA from cultured cells and NPC specimens was extracted using TRIzol reagent (Invitrogen) as previously described [48]. RNA was reverse transcribed using reverse transcriptase (Promega, Madison, WI, USA) with random primers (Promega) for TRIAP1 or Bulge-Loop miRNA specific RT-primers (RiboBio, Guangzhou, China) for miR-320b. Quantitative RT-PCR reactions were performed on a CFX96 Touch sequence detection system (Bio-Rad, Hercules, CA, USA). Using GAPDH or U6 as internal controls for TRIAP1 and miR-320b, respectively, the relative expression levels were calculated by the 2^-ΔΔCT^ method [49].

### Western blotting

Cell lysis was performed at 4°C using RIPA buffer containing a protease inhibitor cocktail (Fdbio Science, Hangzhou, China). Equal amounts of protein were separated on 12% SDS-PAGE gels and transferred to polyvinylidene fluoride membranes (Merck Millipore, Billerica, MA, USA). The membranes were incubated with rabbit polyclonal anti-TRIAP1 antibody (1:200; Santa Cruz Biotechnology, Beverly, MA, USA), followed by incubation with anti-rabbit IgG secondary antibody (1:5000; Epitomics, Burlingame, CA, USA). Anti-α-tubulin antibody (1:1000; Sigma-Aldrich) was used as a protein loading control. Detection was visualized by enhanced chemiluminescence.

### Plasmids, mimic, inhibitor, siRNA and virus constructs

Small interfering RNAs targeting TRIAP1 (siTRIAP1-1 5’-AGGCAUGCACGGACAUGAATT-3’; siTRIAP1-2 5’-GAAAGAGAUUCCUAUUGAATT-3’), miR-320b mimic (5’-AAAAGCUGGGUUGAGAGGGCAA-3’) and miR-320b inhibitor (5’-UUGCCCUCUCAACCCAGCUUUU-3’) were purchased from GenePharma company (Suzhou, China). The human TRIAP1 gene, EBV encoded LMP1 gene and synthesized short hairpin RNA targeting TRIAP1 (shTRIAP1) were cloned into the pSin-EF2- puromycin and pSuper-retro-puromycin vectors, respectively (Addgene, Cambridge, MA, USA). CNE-2 and SUNE-1 cells were transfected with oligonucleotides (100 nM) or plasmids (2 μg) using Lipofectamine 2000 reagent (Invitrogen), and then harvested for assays 48 h after transfection. Stable SUNE-1 cell lines expressing TRIAP1 and shTRIAP1 were generated by lentiviral infection using 293FT cells and selected using 0.5 μg ml^-1^ puromycin.

### MTT, colony-formation and anchorage-independent soft-agar assays

For the MTT assay, 1,000 transfected cells were seeded in 96-well plates and exposed to MTT (BD Biosciences) for 4 h at 1, 2, 3, 4, and 5 days. The absorbance values were measured at 490 nm. For the colony-formation assay, 500 transfected cells were plated in six-well plates and cultured for 7 or 12 days. The colonies were stained with 0.5% crystal violet for quantification after fixation with 4% paraformaldehyde. The anchorage-independent growth assays were performed by soft agar culture of 2.5 × 10^4^ transfected cells in six-well plates for 7 or 12 days. The colony numbers were counted using an inverted microscope.

### Cell apoptosis analysis

The apoptosis assay was performed using the Annexin V-FITC/PI Apoptosis Detection Kit (KeyGEN BioTECH, Nanjing, China). Briefly, 2 to 5 × 10^5^ transfected cells were rinsed twice with PBS and then resuspended in 500 μl of binding buffer, followed by staining with 5 μl of Annexin V-FITC and propidium iodide (PI) for 15 minutes at room temperature in the dark. The detection was performed using a flow cytometer on a Beckman Gallios detection system (Beckman Coulter Inc., CA, USA).

### Mitochondrial and immunofluorescent staining

For mitochondrial staining, transfected cells were grown on coverslips inside a Petri dish (Nest Biotechnology, Wuxi, China) for 24 h and stained with MitoTracker Red CMXRos (0.04 μM for live mitochondrial imaging; 0.4 μM for fixation and permeabilization after mitochondrial staining; ThermoFisher Scientific, Waltham, MA) for 30 minutes at 37°C. After staining, the staining solution was replaced with pre-warmed PBS and live mitochondrial were either observed using a confocal laser-scanning microscope (Olympus FV1000, Tokyo, Japan) or allowed continue to fix and permeabilize. After permeabilization, coverslips were incubated with either rabbit polyclonal anti-TRIAP1 antibody (1:100; Santa Cruz Biotechnology) or mouse monoclonal anti-cytochrome *c* antibody (1:300; Cell Signaling Technology, Danvers, MA) and then incubated with species-matched Alexa Fluor 488 or 594 goat IgG secondary antibody (Life Technologies, Carlsbad, CA, USA). After being counterstained with 4′, 6-diamidino-2-phenylindole (DAPI), cells were imaged using the confocal laser-scanning microscope (Olympus FV1000).

### Flow cytometric analysis of mitochondrial membrane potential

Mitochondrial membrane potentials (△*Ψ*m) were detected using a JC-1 Apoptosis Detection Kit (KeyGEN BioTECH). A total of 2 to 5 × 10^5^ transfected cells were collected, washed twice with PBS, and incubated with 500 μl of prewarmed JC-1 incubation buffer at 37°C for 20 minutes. After incubation, cells were centrifuged, rinsed twice with incubation buffer, and resuspended in 500 μl of incubation buffer. The △*Ψ*m analysis was performed using a flow cytometer on a Beckman Gallios detection system (Beckman Coulter).

### Caspase-3/7 assay

The activity of caspase-3 and caspase-7 was determined using a Caspase-Glo 3/7 Assay Kit (Promega) according to the manufacturer’s instructions. Transfected cells were grown in 100 μl of cultured medium in 96-well plates and equilibrated to room temperature before the assay. A total of 100 μl of Caspase-Glo 3/7 reagent was added to each well and incubated for 1 h in the dark. Luminescence was measured using the luminometer (Promega).

### In vivo tumor growth model, IHC staining and TUNEL assay

Six-week-old male BALB/c nude mice were purchased from the Medical Experimental Animal Center of Guangdong Province (Guangzhou, China). The nude mice were implanted with 1 × 10^6^ SUNE-1 cells stably overexpressing TRIAP1, shTRIAP1 or the corresponding negative control in the dorsal flank. For miR-320b overexpression and inhibition experiments, 1 × 10^6^ SUNE-1 cells were subcutaneously injected into the dorsal flank of nude mice after pre-transfected with agomir-320b, antagomir-320b or NC control (200nM, RiboBio). After 7 days, when the tumor volume reached 100mm^3^, intratumoral injection of agomir-320b (5nM), antagomir-320b (5nM) or NC control (5nM) was performed twice a week for 3 weeks. The weights and tumor volumes were measured twice weekly. The mice were sacrificed 28–35 days after implantation, and the tumors were dissected, weighted and paraffin embedded. Serial sections were subjected to IHC analysis using anti-TRIAP1 antibody or anti-Ki67 antibody (ZSGB-Bio, Beijing, China). A proliferation index was measured using the percentage of positive Ki67 cells. A TUNEL assay was performed on sections from paraffin-embedded mouse specimens using the TUNEL *In situ* Cell Death Detection Kit, Biotin POD (KeyGEN BioTECH) according to manufacturer’s instructions. The apoptotic index was quantified by the proportion of positive TUNEL cells. All animal experiments were approved by the Institutional Animal Care and Use Ethics Committee.

### Dual luciferase reporter assay

Both the conserved (position 265–272) and poor conserved (position 550–556) binding sites were mutated. The mutation (Mt) and wild-type (Wt) versions of the TRIAP1 3′ UTR were generated and cloned into the psiCHECK-2 luciferase reporter plasmid (Promega). Cells were seeded into 6-well plates and co-transfected with the TRIAP1 Wt or Mt 3′ UTR reporter plasmids (2 μg), along with the miR-320b mimics (100 nM) or miR-320b inhibitor (100nM) or miRNA negative control (miR-Ctrl, 100 nM) using Lipofectamine 2000 reagent (Invitrogen). Renilla and firefly luciferase activities were measured 24 h after transfection using the Dual-Luciferase Reporter Assay System (Promega).

### Statistical analysis

All statistical analyses were performed using SPSS 16.0 software (IBM, Armonk, NY, USA). The data are represented as the mean ± SD resulting from at least three independent experiments. The χ^2^ and Fisher’s exact tests were used to compare Categorical variables. Survival curves were calculated using the Kaplan-Meier method, and the differences were compared using a log-rank test. A multivariate analysis using a Cox proportional hazards model was performed to assess independent prognostic factors. The correlation of TRIAP1 and miR-320b mRNA expression was compared using a Pearson’s χ^2^ test. Comparisons between groups were evaluated by two-tailed Student’s *t*-tests. *P* < 0.05 was considered significant.

## Supporting Information

S1 Table Clinicopathological characteristics of studied patients and expression of TRIAP1 and miR-320b in 204 patients with nasopharyngeal carcinoma. (DOC)

S2 Table Correlation between the clinicopathological features and TRIAP1 expression in 204 patients with nasopharyngeal carcinoma. (DOC)

S3 Table Univariate and multivariable Cox regression analysis of prognostic factors in 204 patients with nasopharyngeal carcinoma. (DOC)

S1 Fig Overexpression and knockdown of TRIAP1 expression in CNE-2 and SUNE-1 cells. (**A**) Representative western blotting analysis of TRIAP1 overexpression in CNE-2 and SUNE-1 cells. α-Tubulin served as the loading control. (**B**) Quantitative RT-PCR analysis of TRIAP1 overexpression in CNE-2 and SUNE-1 cells. (**C**) Representative western blotting analysis of TRIAP1 knockdown in CNE-2 and SUNE-1 cells. α-Tubulin served as the loading control. (**D**) Quantitative RT-PCR analysis of TRIAP1 knockdown in CNE-2 and SUNE-1 cells. Each experiment was independently repeated at least three times. The data are presented as the mean ± s.d. Student’s *t*-test, * *P* < 0.05, ** *P* < 0.01, *** *P* < 0.001.
(TIF)

S2 Fig Knockdown of TRIAP1 enhances mitochondrial fragmentation. (**A**) Representative images of live mitochondria for CNE-2 and SUNE-1 cells transfected with siSCR, siTRIAP1-1 or siTRIAP1-2 after staining with MitoTracker Red. Scale bar, 10 μm.
(TIF)

S3 Fig Candidate miRNAs target TRIAP1. Candidate miRNAs predicted by three different bioinformatics algorithms, TargetScan, miRanda and miRWalk. Predicted miRNAs are intersected with 33 downregulated miRNAs in nasopharyngeal carcinoma compared with normal nasopharyngeal tissues published in previous miRNA microarray data (NCBI/GEO/GSE32960, n = 330, including 312 NPC tissues and 18 normal nasopharyngeal tissues).
(TIF)

S4 Fig TRIAP1 mediates the effects of miR-320b on NPC cell apoptosis. (**A**) Representative dot plots of flow cytometric analyses of TRIAP1 knockdown in CNE-2 and SUNE-1 cells subjected to Annexin V and propidium iodide (PI) staining. Each experiment was independently repeated at least three times. (**B**) Representative dot plots of mitochondrial membrane potential of TRIAP1 knockdown in CNE-2 and SUNE-1 cells subjected to JC-1 staining. The percentage of cells with FL1-positive and FL2-negative signals represents depolarized mitochondrial cells. Each experiment was independently repeated at least three times.
(TIF)

S5 Fig Inhibition of TRIAP1 abrogates the effects of miR-320b downregulation on NPC cell proliferation and mitochondrial apoptosis. (**A-F**) CNE-2 and SUNE-1 cells were co-transfected with a miR-320b inhibitor or inhibitor-Ctrl and either siSCR or siRNA targeting TRIAP1. (**A**) MTT assay showing that inhibition of TRIAP1 abrogates the promoted effects of miR-320b on cell proliferation. (**B-F**) Flow cytometric analysis (**B-E**) and caspase-3/7 (**F**) assays showing that TRIAP1 inhibition reverses the inhibiting effects of miR-320b on mitochondrial membrane depolarization and apoptosis. Each experiment was independently repeated at least three times. The data are presented as the mean ± s.d. Student’s *t*-test, * *P* < 0.05, ** *P* < 0.01, *** *P* < 0.001.
(TIF)

S6 Fig TRIAP1 mediates the effects of miR-320b on cytochrome c release. **A** and **B,** Representative images of mitochondria, cytochrome *c* and TRIAP1 subcellular locations for CNE-2 (**A**) and SUNE-1 (**B**) cells transiently transfected with siSCR, siTRIAP1-1 or siTRIAP1-2 after being stained with cytochrome *c* and TRIAP1 primary antibodies. Scale bar, 10 μm. Each experiment was independently repeated at least three times.
(TIF)

S7 Fig miR-320b was significantly downregulated in nasopharyngeal carcinoma. (**A**) miR-320b was significantly downregulated in nasopharyngeal carcinoma compared with normal nasopharyngeal tissues published in previous miRNA microarray data (NCBI/GEO/GSE32960, n = 330, including 312 NPC tissues and 18 normal nasopharyngeal tissues).
(TIF)

S8 Fig Combination of miR-320b and TRIAP1 expression correlates with poor survival in NPC patients. **A** and **B**, Kaplan-Meier analysis of overall survival (**A**) and disease-free survival (**B**) for 204 NPC patients with combined miR-320b and TRIAP1 expression showing that patients with high TRIAP1 expression and low miR-320b expression have the worst survival. *P* value was determined by the log-rank test.
(TIF)

S9 Fig Expression of miR-320b in xenograft assays. (**A)** miR-320b expression was detected in xenograft tumor tissues after intratumor injection of either agomir-320b, antagomir-320b or NC control by quantitative RT-PCR. The data are presented as the mean ± s.d. Student’s *t*-test, * *P* < 0.05.
(TIF)

S10 Fig Regulation of TRIAP1 in LMP1/TP53/p21 pathway. (**A**) TRIAP1 and p21 protein expression by western blotting in CNE-2 and SUNE-1 cells transfected with scrambled siRNA control (siSCR) or TRIAP1-specific siRNA. (**B**) LMP1, TP53, TRIAP1 and p21 protein expression by western blotting in SUNE-1 cells transfected with the empty psin-EF2 vector control (Vector) or psin-EF2-LMP1 plasmid (LMP1) overexpressing LMP1. Each experiment was independently repeated at least three times.
(TIF)
