# Supplementary material for: Overexpression of Mitochondria Mediator Gene TRIAP1 by miR-320b Loss Is Associated with Progression in Nasopharyngeal Carcinoma
Source: PLoS Genet. 2016 Jul 18;12(7):e1006183. doi: 10.1371/journal.pgen.1006183 (PMC4948882; doi:10.1371/journal.pgen.1006183)
Supplement: S1 Table — (DOC) [file pgen.1006183.s001.doc]

**Supplementary information, Table S1**: Clinicopathological characteristics of studied patients and expression of TRIAP1 and miR-320b in 204 patients with nasopharyngeal carcinoma

| **Characteristic** | **No. of patients** | **(%)** |
| --- | --- | --- |
| **Age** |  |  |
| ≤ 45 years | 104 | 51.0 |
| > 45 years | 100 | 49.0 |
| **Gender** |  |  |
| Male | 155 | 76.0 |
| Female | 49 | 24.0 |
| **WHO Type** |  |  |
| IIa | 8 | 3.9 |
| IIb | 196 | 96.1 |
| **VCA-IgA** |  |  |
| < 1:80 | 22 | 10.8 |
| ≥ 1:80 | 182 | 89.2 |
| **EA-IgA** |  |  |
| < 1:10 | 41 | 20.1 |
| ≥ 1:10 | 163 | 79.9 |
| **T Stage** |  |  |
| T1-T2 | 83 | 40.7 |
| T3-T4 | 121 | 59.3 |
| **N Stage** |  |  |
| N0-N1 | 127 | 84.3 |
| N2-N3 | 77 | 15.7 |
| **TNM Stage** |  |  |
| I-II | 55 | 27.0 |
| III-IV | 149 | 73.0 |
| **Locoregional failure** |  |  |
| No | 172 | 84.3 |
| Yes | 32 | 15.7 |
| **Distant metastasis** |  |  |
| No | 171 | 83.8 |
| Yes | 33 | 16.2 |
| **Death** |  |  |
| No | 156 | 76.5 |
| Yes | 48 | 23.5 |
| **Expression of TRIAP1** |  |  |
| Low expression | 108 | 52.9 |
| High expression | 96 | 47.1 |
| **Expression of miR-320b** |  |  |
| Low expression | 102 | 50 |
| High expression | 102 | 50 |

Abbreviations: WHO type IIa, differentiated non-keratinizing nasopharyngeal carcinoma; WHO type IIb, undifferentiated non-keratinizing nasopharyngeal carcinoma; VCA-IgA, viral capsid antigen immunoglobulin A; EA-IgA, early antigen immunoglobulin A. All patients were restaged according to the 7^th^ edition of the AJCC Cancer Staging Manual.
