# Supplementary material for: Overexpression of Mitochondria Mediator Gene TRIAP1 by miR-320b Loss Is Associated with Progression in Nasopharyngeal Carcinoma
Source: PLoS Genet. 2016 Jul 18;12(7):e1006183. doi: 10.1371/journal.pgen.1006183 (PMC4948882; doi:10.1371/journal.pgen.1006183)
Supplement: S2 Table — (DOC) [file pgen.1006183.s002.doc]

**Supplementary information, Table S2**: Correlation between the clinicopathological features and TRIAP1 expression in 204 patients with nasopharyngeal carcinoma

| **Characteristic** | **No. of patients** | **TRIAP1 expression** | | ***P* Value*** |
| --- | --- | --- | --- | --- |
|  |  | **Low, *n* (%)** | **High, *n* (%)** |  |
| **Age** |  |  |  |  |
| ≤ 45 years | 104 | 54 (50.0) | 50 (52.1) | 0.781 |
| > 45 years | 100 | 54 (50.0) | 46 (47.9) |  |
| **Gender** |  |  |  |  |
| Male | 155 | 79 (73.1) | 76 (79.2) | 0.330 |
| Female | 49 | 29 (26.9) | 20 (20.8) |  |
| **WHO Type** |  |  |  |  |
| IIa | 8 | 4 (3.7) | 4 (4.2) | 1.000 |
| IIb | 196 | 104 (96.3) | 92 (95.8) |  |
| **VCA-IgA** |  |  |  |  |
| < 1:80 | 22 | 15 (13.9) | 7 (7.3) | 0.175 |
| ≥ 1:80 | 182 | 93 (86.1) | 89 (92.7) |  |
| **EA-IgA** |  |  |  |  |
| < 1:10 | 41 | 22 (20.4) | 19 (19.8) | 1.000 |
| ≥ 1:10 | 163 | 86 (79.6) | 77 (80.2) |  |
| **T Stage** |  |  |  |  |
| T1-T2 | 83 | 49 (45.4) | 34 (35.4) | 0.156 |
| T3-T4 | 121 | 59 (54.6) | 62 (64.6) |  |
| **N Stage** |  |  |  |  |
| N0-N1 | 127 | 65 (60.2) | 62 (64.6) | 0.564 |
| N2-N3 | 77 | 43 (39.8) | 34 (35.4) |  |
| **TNM Stage** |  |  |  |  |
| I-II | 55 | 31 (28.7) | 24 (25.0) | 0.636 |
| III-IV | 149 | 77 (71.3) | 72 (75.0) |  |
| **Locoregional failure** |  |  |  |  |
| No | 172 | 94 (87.0) | 78 (81.2) | 0.335 |
| Yes | 32 | 14 (13.0) | 18 (18.8) |  |
| **Distant metastasis** |  |  |  |  |
| No | 171 | 100 (92.6) | 71 (74.0) | **<0.001** |
| Yes | 33 | 8 (7.4) | 25 (26.0) |  |
| **Death** |  |  |  |  |
| No | 156 | 92 (85.2) | 64 (66.7) | **0.003** |
| Yes | 48 | 16 (14.8) | 32 (33.3) |  |

Abbreviations: WHO type IIa, differentiated non-keratinizing nasopharyngeal carcinoma; WHO type IIb, undifferentiated non-keratinizing nasopharyngeal carcinoma; VCA-IgA, viral capsid antigen immunoglobulin A; EA-IgA, early antigen immunoglobulin A. All patients were restaged according to the 7^th^ edition of the AJCC Cancer Staging Manual. Bold values indicate *P* < 0.05, *P* value is determined by χ2 and Fisher’s exact tests.
