# Supplementary material for: Overexpression of Mitochondria Mediator Gene TRIAP1 by miR-320b Loss Is Associated with Progression in Nasopharyngeal Carcinoma
Source: PLoS Genet. 2016 Jul 18;12(7):e1006183. doi: 10.1371/journal.pgen.1006183 (PMC4948882; doi:10.1371/journal.pgen.1006183)
Supplement: S3 Table — (DOC) [file pgen.1006183.s003.doc]

**Supplementary information Table S3**: Univariate and multivariable Cox regression analysis of prognostic factors in 204 patients with nasopharyngeal carcinoma

| **Variable** | **Univariate analysis** | | |  | **Multivariate analysis** | | | |
| --- | --- | --- | --- | --- | --- | --- | --- | --- |
|  | **HR** | **95%CI** | ***P-*value** |  | **HR** | **95%CI** | ***P-*value** | |
| **Overall survival** | | | | | | | | |
| TRIAP1 expression (High vs. low) | 2.63 | 1.44-4.80 | **0.002** |  | 2.75 | 1.50-5.03 | | **0.001** |
| TNM stage (III-IV vs. I-II) | 4.73 | 1.70-13.18 | **0.003** |  | 4.07 | 1.46-11.36 | | **0·007** |
| Age (≥ 45 vs. < 45 years) | 2.11 | 1.17-3.82 | **0.013** |  | 2.16 | 1.19-3.93 | | **0.011** |
| Gender (Male vs. female) | 3.05 | 1.21-7.70 | **0.018** |  | 2.60 | 1.03-6.59 | | **0.043** |
| WHO type (IIa vs. IIb) | 2.66 | 0.95-7.41 | 0.06 |  |  |  | |  |
| VCA IgA (≥ 1:80 vs. < 1:80) | 2.96 | 0.71-12.21 | 0.13 |  |  |  | |  |
| EA IgA (≥ 1:10 vs. < 1:10) | 1.15 | 0.56-2.37 | 0.71 |  |  |  | |  |
| **Disease-free survival** | | | | | | | | |
| TRIAP1 expression (High vs. low) | 2.54 | 1.48-4.37 | **0.001** |  | 2.54 | 1.47-4.38 | | **<0.001** |
| Age (≥ 45 vs. < 45 years) | 1.74 | 1.03-2.94 | **0.039** |  | 1.81 | 1.07-3.08 | | **0.028** |
| Gender (Male vs. female) | 2.57 | 1.17-5.67 | **0.019** |  | 2.27 | 1.03-5.02 | | **0.042** |
| TNM stage (III-IV vs. I-II) | 2.31 | 1.13-4.70 | **0.021** |  | 2.20 | 0.99-4.13 | | 0.052 |
| WHO type (IIa vs. IIb) | 2.21 | 0.80-6.10 | 0.13 |  |  |  | |  |
| VCA IgA (≥ 1:80 vs. < 1:80) | 2.45 | 0.77-7.84 | 0.13 |  |  |  | |  |
| EA IgA (≥ 1:10 vs. < 1:10) | 1.28 | 0.65-2.52 | 0.48 |  |  |  | |  |

Abbreviations: WHO type IIa, differentiated non-keratinizing nasopharyngeal carcinoma; WHO type IIb, undifferentiated non-keratinizing nasopharyngeal carcinoma; VCA-IgA, viral capsid antigen immunoglobulin A; EA-IgA, early antigen immunoglobulin A; HR, hazard ratio; NS, not significant. Bold values indicate *P* < 0.05, *P* value wss determined by Cox regression analysis.
