# Supplementary figures and images for: Overexpression of Mitochondria Mediator Gene TRIAP1 by miR-320b Loss Is Associated with Progression in Nasopharyngeal Carcinoma
Source: PLoS Genet. 2016 Jul 18;12(7):e1006183. doi: 10.1371/journal.pgen.1006183 (PMC4948882; doi:10.1371/journal.pgen.1006183)

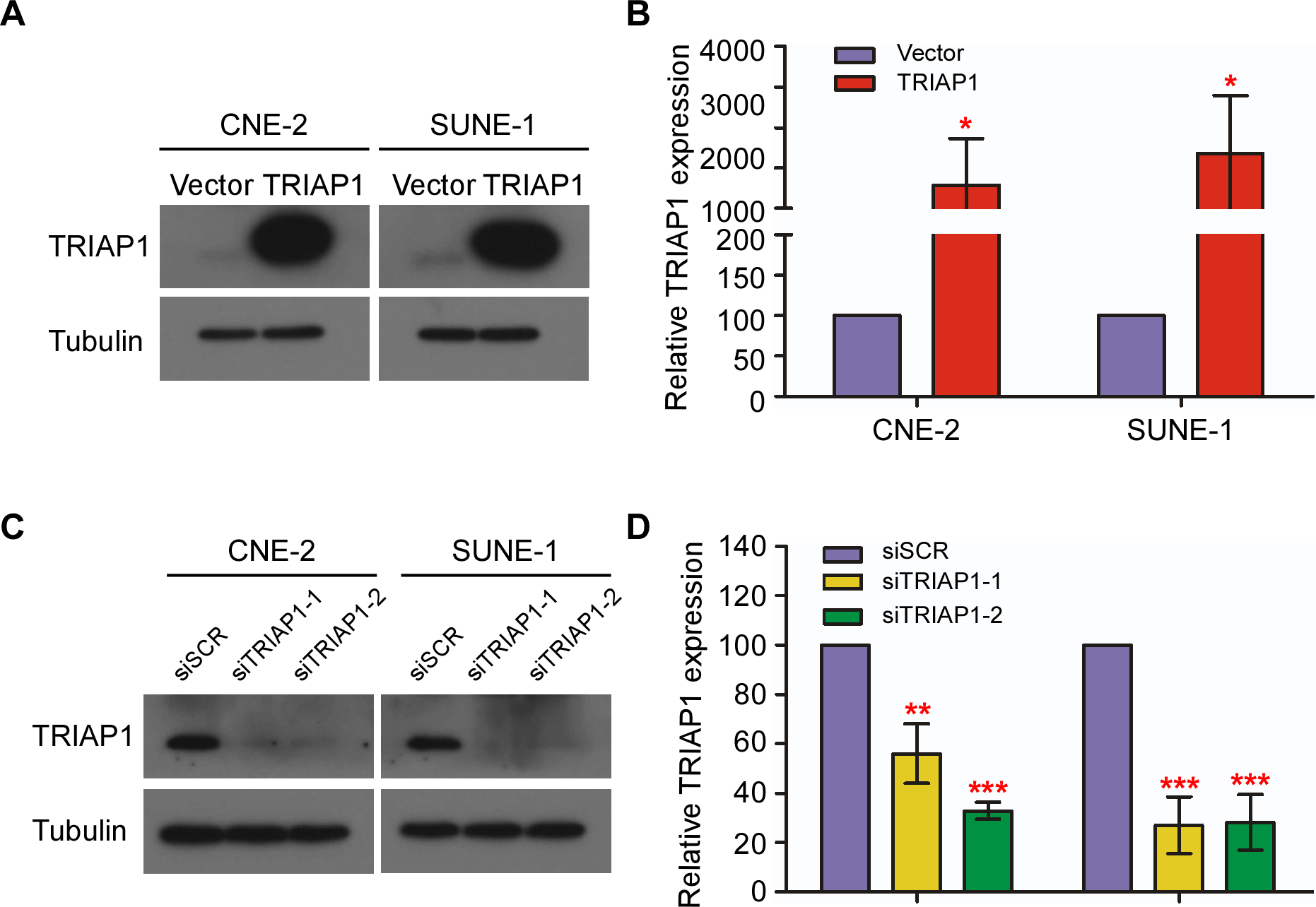

Supplement: S1 Fig — (A) Representative western blotting analysis of TRIAP1 overexpression in CNE-2 and SUNE-1 cells. α-Tubulin served as the loading control. (B) Quantitative RT-PCR analysis of TRIAP1 overexpression in CNE-2 and SUNE-1 cells. (C) Representative western blotting analysis of TRIAP1 knockdown in CNE-2 and SUNE-1 cells. α-Tubulin served as the loading control. (D) Quantitative RT-PCR analysis of TRIAP1 knockdown in CNE-2 and SUNE-1 cells. Each experiment was independently repeated at least three times. The data are presented as the mean ± s.d. Student’s t-test, * P < 0.05, ** P < 0.01, *** P < 0.001. (TIF) [file pgen.1006183.s004.tif]

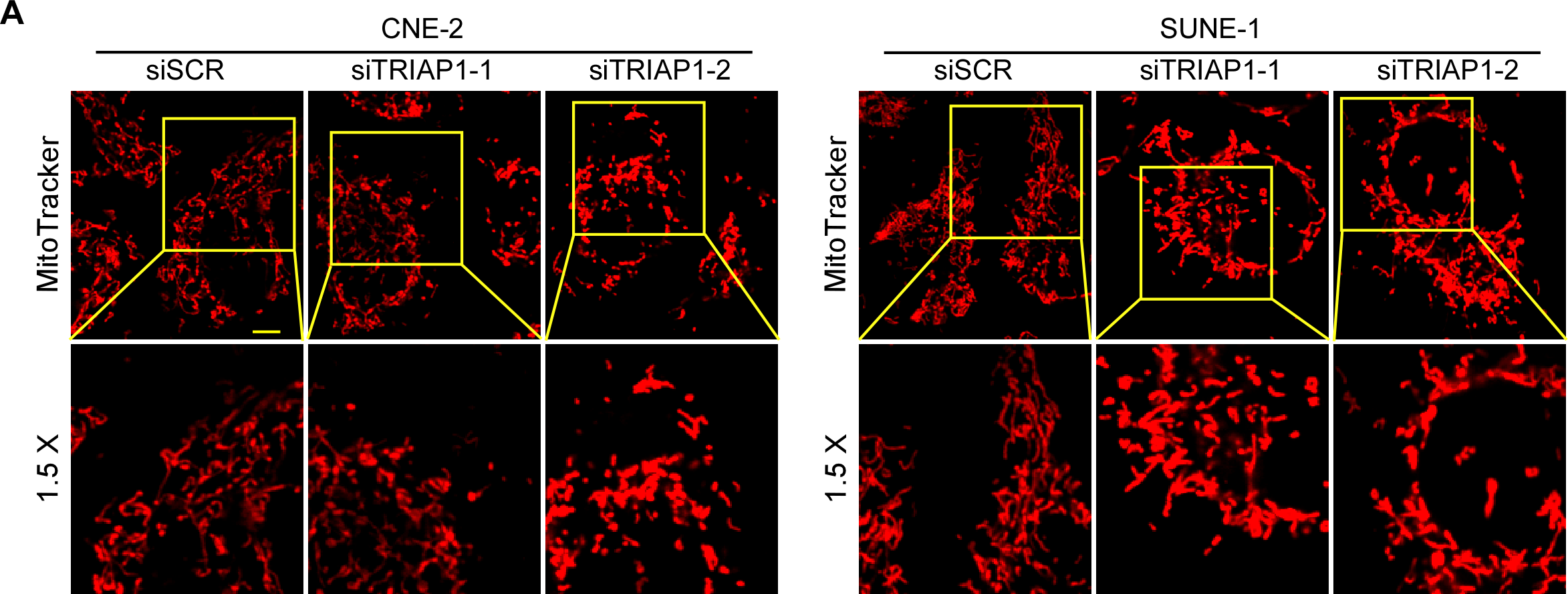

Supplement: S2 Fig — (A) Representative images of live mitochondria for CNE-2 and SUNE-1 cells transfected with siSCR, siTRIAP1-1 or siTRIAP1-2 after staining with MitoTracker Red. Scale bar, 10 μm. (TIF) [file pgen.1006183.s005.tif]

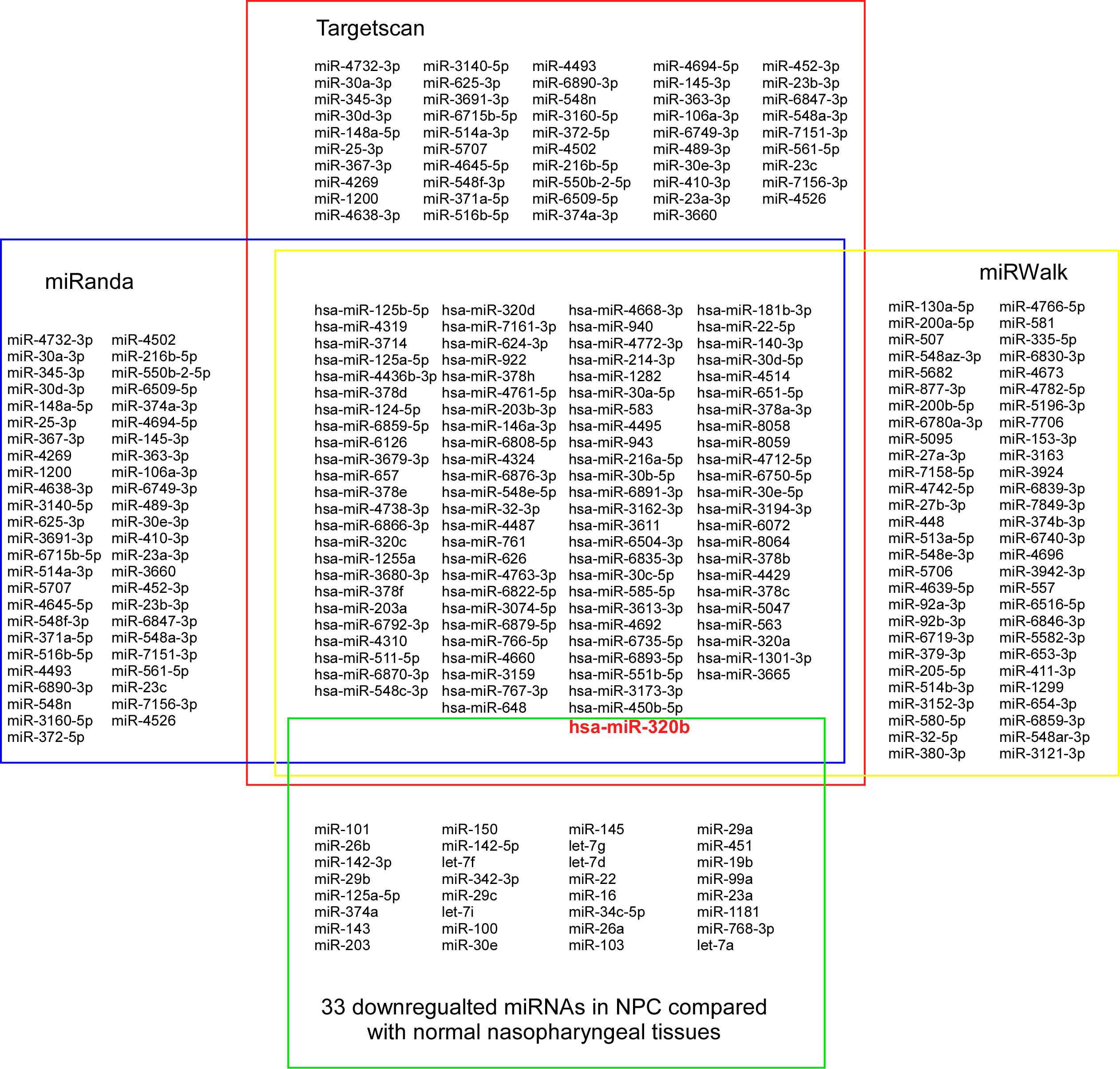

Supplement: S3 Fig — Candidate miRNAs predicted by three different bioinformatics algorithms, TargetScan, miRanda and miRWalk. Predicted miRNAs are intersected with 33 downregulated miRNAs in nasopharyngeal carcinoma compared with normal nasopharyngeal tissues published in previous miRNA microarray data (NCBI/GEO/GSE32960, n = 330, including 312 NPC tissues and 18 normal nasopharyngeal tissues). (TIF) [file pgen.1006183.s006.tif]

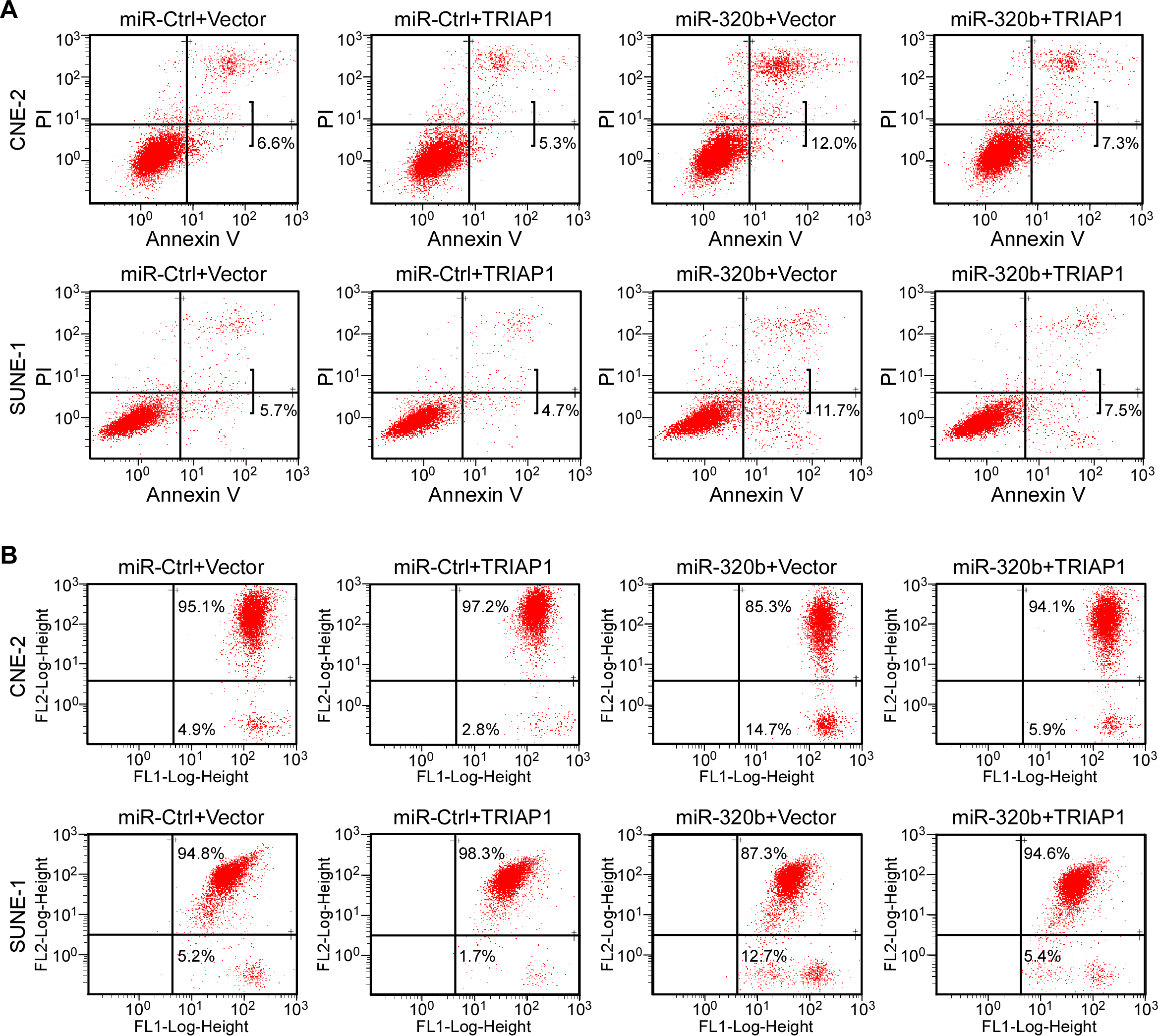

Supplement: S4 Fig — (A) Representative dot plots of flow cytometric analyses of TRIAP1 knockdown in CNE-2 and SUNE-1 cells subjected to Annexin V and propidium iodide (PI) staining. Each experiment was independently repeated at least three times. (B) Representative dot plots of mitochondrial membrane potential of TRIAP1 knockdown in CNE-2 and SUNE-1 cells subjected to JC-1 staining. The percentage of cells with FL1-positive and FL2-negative signals represents depolarized mitochondrial cells. Each experiment was independently repeated at least three times. (TIF) [file pgen.1006183.s007.tif]

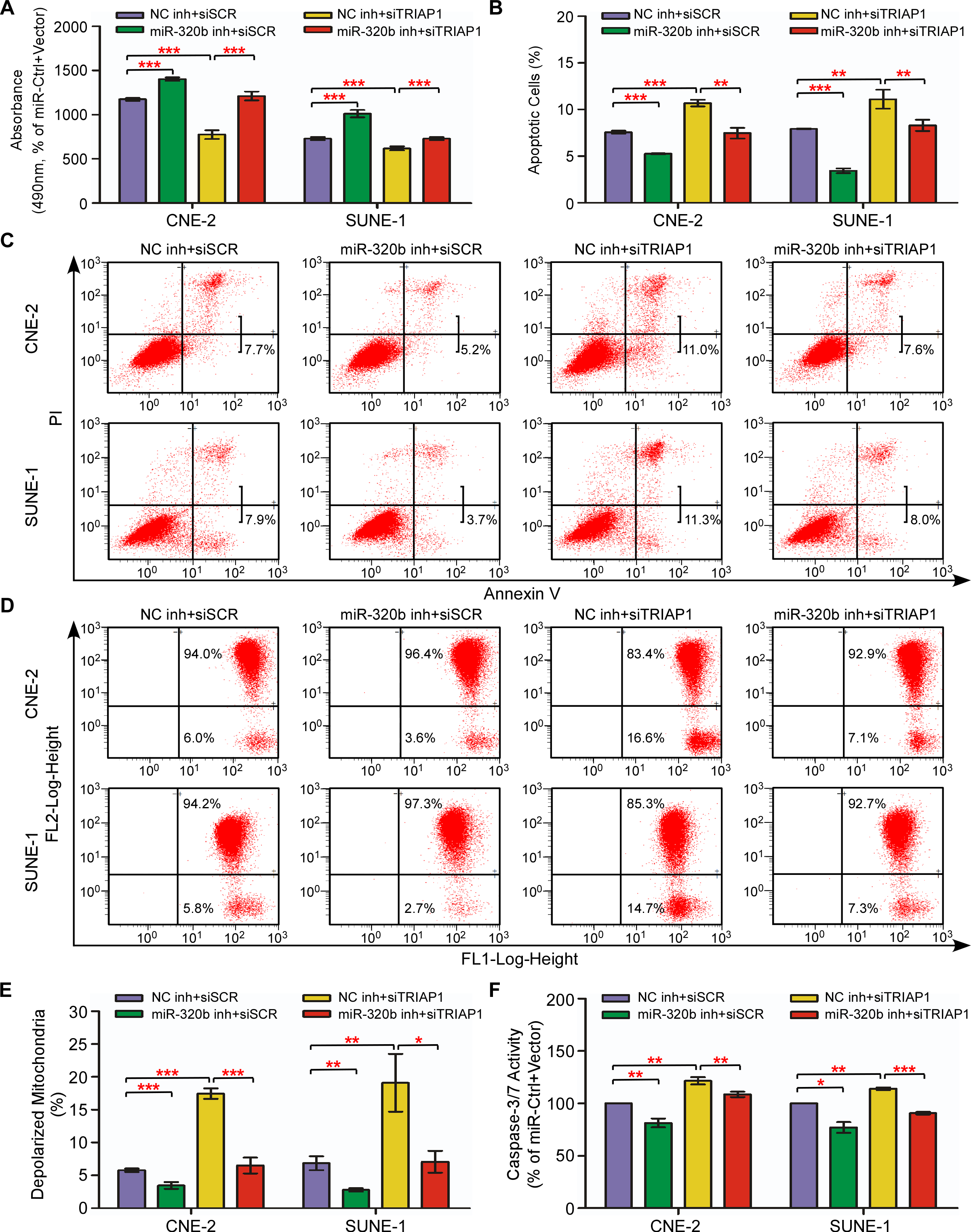

Supplement: S5 Fig — (A-F) CNE-2 and SUNE-1 cells were co-transfected with a miR-320b inhibitor or inhibitor-Ctrl and either siSCR or siRNA targeting TRIAP1. (A) MTT assay showing that inhibition of TRIAP1 abrogates the promoted effects of miR-320b on cell proliferation. (B-F) Flow cytometric analysis (B-E) and caspase-3/7 (F) assays showing that TRIAP1 inhibition reverses the inhibiting effects of miR-320b on mitochondrial membrane depolarization and apoptosis. Each experiment was independently repeated at least three times. The data are presented as the mean ± s.d. Student’s t-test, * P < 0.05, ** P < 0.01, *** P < 0.001. (TIF) [file pgen.1006183.s008.tif]

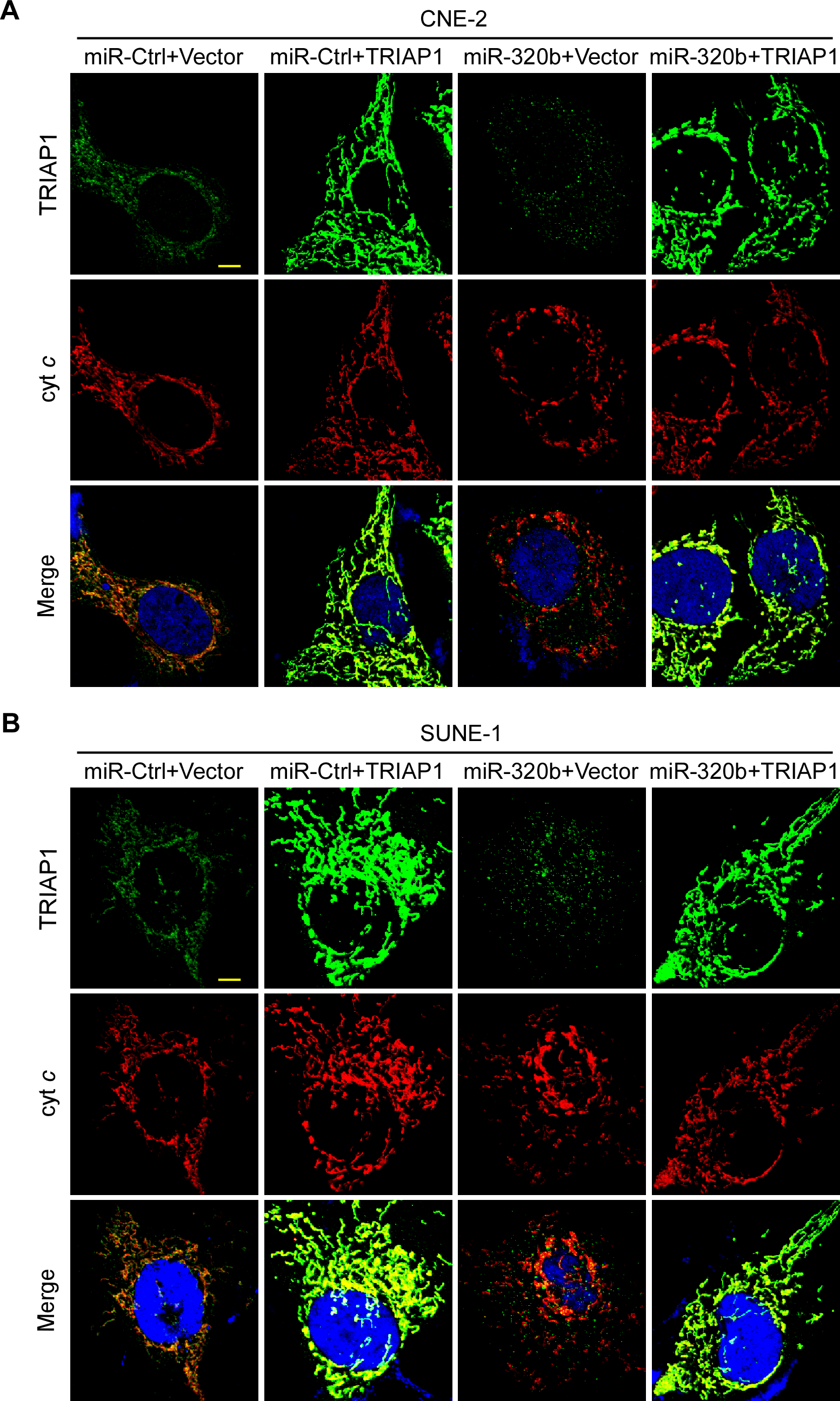

Supplement: S6 Fig — A and B, Representative images of mitochondria, cytochrome c and TRIAP1 subcellular locations for CNE-2 (A) and SUNE-1 (B) cells transiently transfected with siSCR, siTRIAP1-1 or siTRIAP1-2 after being stained with cytochrome c and TRIAP1 primary antibodies. Scale bar, 10 μm. Each experiment was independently repeated at least three times. (TIF) [file pgen.1006183.s009.tif]

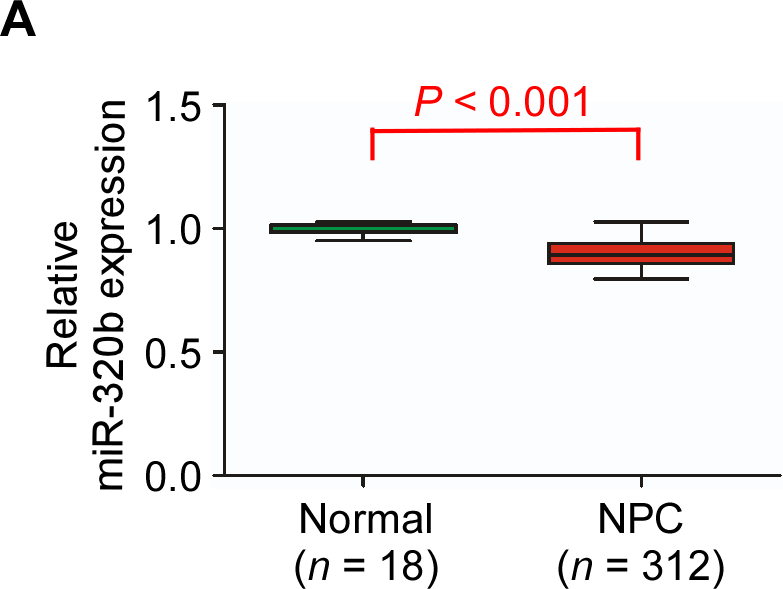

Supplement: S7 Fig — (A) miR-320b was significantly downregulated in nasopharyngeal carcinoma compared with normal nasopharyngeal tissues published in previous miRNA microarray data (NCBI/GEO/GSE32960, n = 330, including 312 NPC tissues and 18 normal nasopharyngeal tissues). (TIF) [file pgen.1006183.s010.tif]

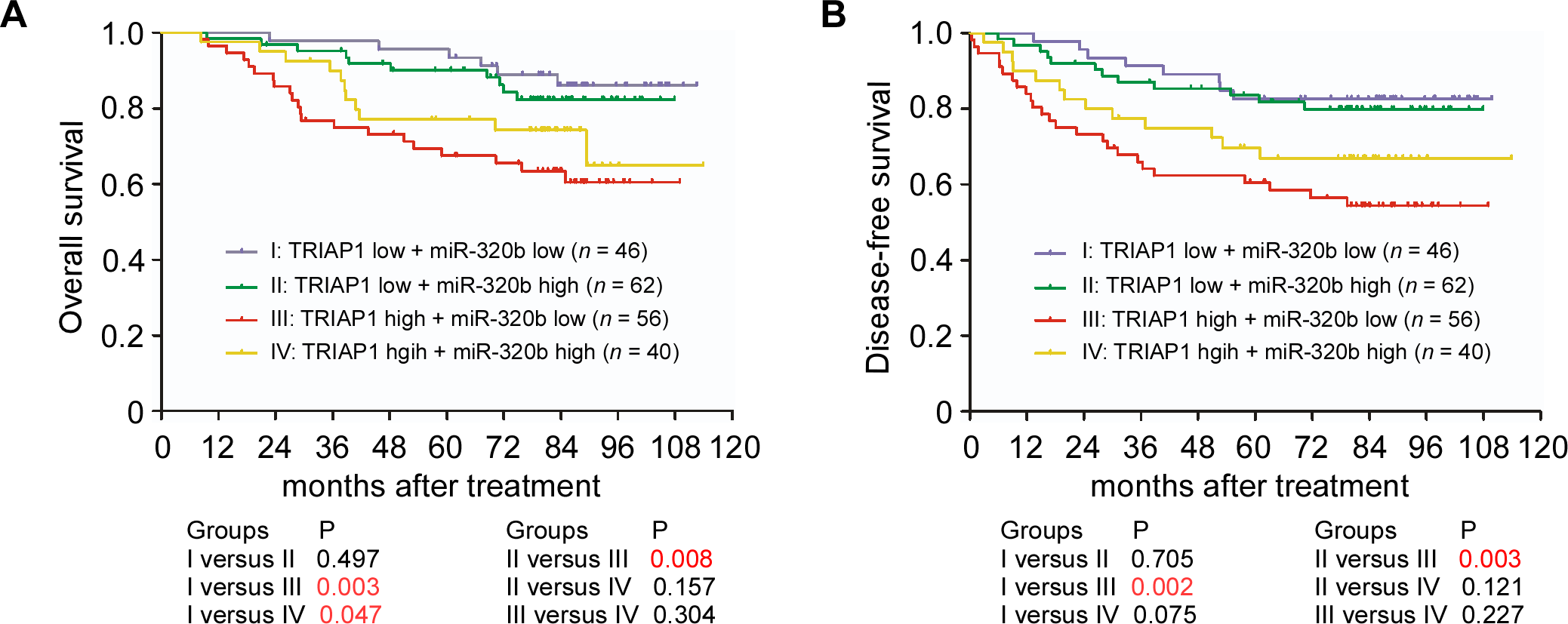

Supplement: S8 Fig — A and B, Kaplan-Meier analysis of overall survival (A) and disease-free survival (B) for 204 NPC patients with combined miR-320b and TRIAP1 expression showing that patients with high TRIAP1 expression and low miR-320b expression have the worst survival. P value was determined by the log-rank test. (TIF) [file pgen.1006183.s011.tif]

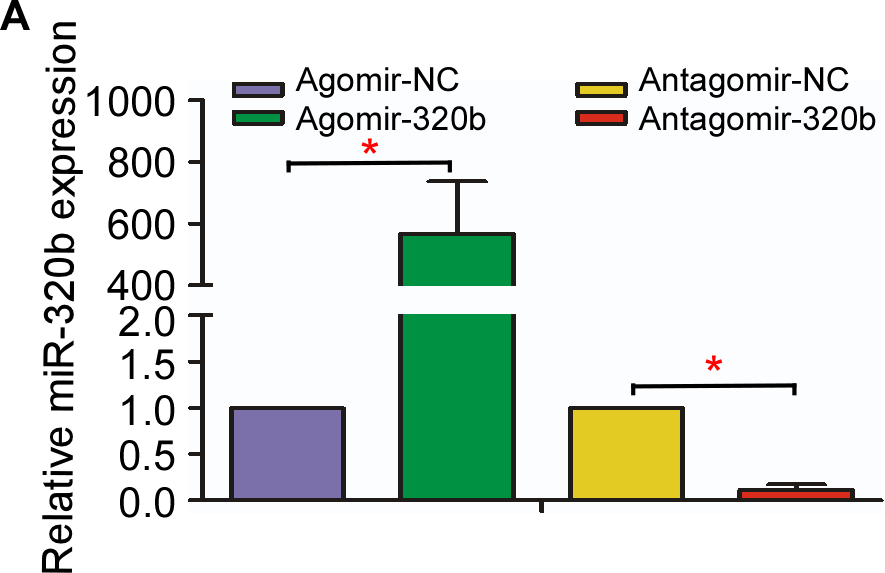

Supplement: S9 Fig — (A) miR-320b expression was detected in xenograft tumor tissues after intratumor injection of either agomir-320b, antagomir-320b or NC control by quantitative RT-PCR. The data are presented as the mean ± s.d. Student’s t-test, * P < 0.05. (TIF) [file pgen.1006183.s012.tif]

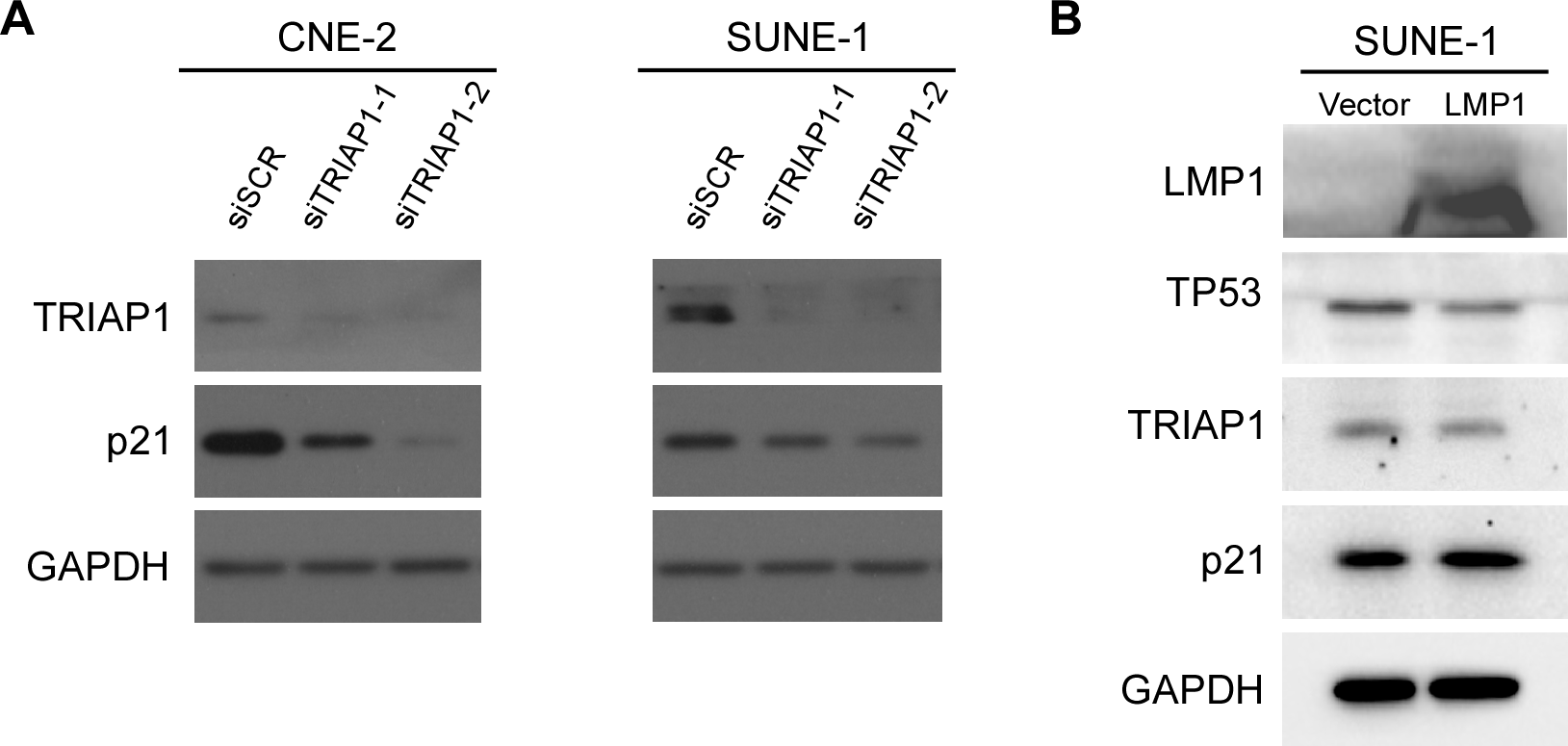

Supplement: S10 Fig — (A) TRIAP1 and p21 protein expression by western blotting in CNE-2 and SUNE-1 cells transfected with scrambled siRNA control (siSCR) or TRIAP1-specific siRNA. (B) LMP1, TP53, TRIAP1 and p21 protein expression by western blotting in SUNE-1 cells transfected with the empty psin-EF2 vector control (Vector) or psin-EF2-LMP1 plasmid (LMP1) overexpressing LMP1. Each experiment was independently repeated at least three times. (TIF) [file pgen.1006183.s013.tif]
